# Interactive Remote Patient Monitoring Devices for Managing Chronic Health Conditions: Systematic Review and Meta-analysis

**DOI:** 10.2196/35508

**Published:** 2022-11-03

**Authors:** Donato Giuseppe Leo, Benjamin J R Buckley, Mahin Chowdhury, Stephanie L Harrison, Masoud Isanejad, Gregory Y H Lip, David J Wright, Deirdre A Lane, the TAILOR investigators

**Affiliations:** 1 Department of Cardiovascular and Metabolic Medicine Institute of Life Course and Medical Sciences, Faculty of Health and Life Sciences University of Liverpool Liverpool United Kingdom; 2 Liverpool Centre for Cardiovascular Science University of Liverpool and Liverpool Heart & Chest Hospital Liverpool United Kingdom; 3 School of Medicine University of Liverpool Liverpool United Kingdom; 4 Department of Musculoskeletal Ageing Institute of Life Course and Medical Sciences, Faculty of Health and Life Sciences University of Liverpool Liverpool United Kingdom; 5 See acknowledgements

**Keywords:** chronic condition, telemonitoring, telemedicine, eHealth, self-monitoring, systematic review, meta-analysis

## Abstract

**Background:**

Telemedicine is an expanding and feasible approach to improve medical care for patients with long-term conditions. However, there is a poor understanding of patients’ acceptability of this technology and their rate of uptake.

**Objective:**

The aim of this study was to systematically review the current evidence on telemonitoring in the management of patients with long-term conditions and evaluate the patients’ uptake and acceptability of this technology.

**Methods:**

MEDLINE, Scopus, and CENTRAL (the Cochrane Central Register of Controlled Trials) were searched from the date of inception to February 5, 2021, with no language restrictions. Studies were eligible for inclusion if they reported any of the following outcomes: intervention uptake and adherence; study retention; patient acceptability, satisfaction, and experience using the intervention; changes in physiological values; all-cause and cardiovascular-related hospitalization; all-cause and disease-specific mortality; patient-reported outcome measures; and quality of life. In total, 2 reviewers independently assessed the articles for eligibility.

**Results:**

A total of 96 studies were included, and 58 (60%) were pooled for the meta-analyses. Meta-analyses showed a reduction in mortality (risk ratio=0.71, 95% CI 0.56-0.89; *P*=.003; *I*^2^=0%) and improvements in blood pressure (mean difference [MD]=−3.85 mm Hg, 95% CI −7.03 to −0.68; *P*=.02; *I*^2^=100%) and glycated hemoglobin (MD=−0.33, 95% CI −0.57 to −0.09; *P*=.008; *I*^2^=99%) but no significant improvements in quality of life (MD=1.45, 95% CI −0.10 to 3; *P*=.07; *I*^2^=80%) and an increased risk of hospitalization (risk ratio=1.02, 95% CI 0.85-1.23; *P*=.81; *I*^2^=79%) with telemonitoring compared with usual care. A total of 12% (12/96) of the studies reported adherence outcomes, and 9% (9/96) reported on satisfaction and acceptance outcomes; however, heterogeneity in the assessment methods meant that a meta-analysis could not be performed.

**Conclusions:**

Telemonitoring is a valid alternative to usual care, reducing mortality and improving self-management of the disease, with patients reporting good satisfaction and adherence. Further studies are required to address some potential concerns regarding higher hospitalization rates and a lack of positive impact on patients’ quality of life.

**Trial Registration:**

PROSPERO CRD42021236291; https://www.crd.york.ac.uk/prospero/display_record.php?RecordID=236291

## Introduction

### Background

In the United Kingdom, 15 million people live with at least one long-term condition [1], with their care accounting for 70% of the National Health Service budget [1]. Those with long-term conditions have significantly reduced quality of life (QoL) as well as an increased risk of morbidity and mortality [2,3]. Cardiovascular disease, diabetes mellitus, and chronic obstructive pulmonary disease (COPD) are the most common chronic conditions worldwide [4]. Lack of care coordination [5,6] and care planning consultation [5,6] are among the common barriers that patients with long-term conditions face. In addition, the restrictions induced by the COVID-19 pandemic have amplified the challenges that people living with chronic diseases experience in terms of managing their health and accessing health care [7].

Advances in technology have the potential to support patients with long-term conditions in managing their health at home, making the provision of remote health care more accessible and efficient [8]. Web-based health care and telemedicine include the remote delivery of care using communication technology (eg, videoconference software, web-based applications, and home-based health measurement) to enable consultations between patients and their care team, providing continuous monitoring of relevant health parameters. This allows health care professionals to promptly respond to changes in patient health status and adapt their clinical management in real time [9].

### Objectives

Recent evidence has deemed telemedicine feasible for patients with long-term conditions and effective in terms of improving medical care [10]. As telemedicine is a rapidly expanding and changing field, recent umbrella reviews [10,11] that consider older primary studies have potentially made conclusions based on noncontemporary data. Therefore, the aim of this systematic review was to update and expand the current literature on telemonitoring by better defining the interventions included to encompass the role that interactive, 2-way communication devices play in improving the care of patients with long-term conditions, as well as evaluate patient uptake and acceptability of this technology.

## Methods

### Overview

This systematic review was registered on PROSPERO (International Prospective Register of Systematic Reviews; CRD42021236291) and conducted in accordance with the PRISMA (Preferred Reporting Items for Systematic Reviews and Meta-Analyses) guidelines [12].

This review aimed to address the following research questions: (1) What is the rate of uptake, patient retention, and patient satisfaction when using 2-way (patient-health care provider) remote patient monitoring devices to manage chronic health conditions? (2) What factors are associated with patient retention and satisfaction when using 2-way (patient-health care provider) remote patient monitoring devices to manage chronic health conditions? (3) Does the use of 2-way (patient-health care provider) remote patient monitoring devices for the management of chronic health conditions affect patient outcomes (eg, changes in physiological measurements, QoL, all-cause and cardiovascular-related hospitalizations, and all-cause and disease-specific mortality)?

### Criteria for Considering Studies to Include in the Review

Studies carried out in any setting aiming to evaluate telemonitoring interventions for participants with at least one chronic condition among the following—cardiovascular disease, COPD, or diabetes mellitus—were eligible for inclusion. All randomized controlled trials (RCTs) and nonrandomized trials, before-and-after (pre-post) studies, and interrupted time series were considered for inclusion. Cross-sectional studies and case reports were excluded. Qualitative studies were included to assess participant satisfaction. Ongoing studies (if any) were also considered and presented in a dedicated table.

### Participants

Adult participants (aged ≥18 years) were eligible for inclusion in this review if they reported one or more of the following chronic health conditions: cardiovascular diseases (eg, coronary artery disease, atrial fibrillation, stroke, heart failure, and hypertension), COPD, or diabetes mellitus.

### Intervention

Interventions designed to remotely collect health information from patients using digital technologies and electronically transfer the information to health care professionals for monitoring and assessment were eligible for inclusion. Only interventions where the participant received a digital device for remote patient monitoring and the participant or their caregiver took physiological measurements and either input the information into the device or the device automatically uploaded the data were included. Health devices suitable for inclusion had to transmit data to the participant’s health care team, and the participant’s health care team had to monitor the information received, assessing it and making appropriate changes to the participant’s treatment accordingly. A 2-way exchange of information was required for a study to be included.

### Comparator

Studies in which usual care or a different intervention was used as control or comparator were also considered as eligible for inclusion, as were studies that did not have a control group.

### Outcomes

The primary outcomes of interest were (1) intervention uptake (number of people willing to participate in the intervention) and adherence (level of commitment of the patient to the prescribed intervention); (2) study retention (number of people who completed the intervention); and (3) patient acceptability (level of acceptance of the intervention by the participants), satisfaction (number of participants pleased with the intervention), and experience using the intervention. Secondary outcomes included (1) changes in physiological measurements (eg, oxygen saturation, blood pressure [BP], and blood glucose level); (2) all-cause and cardiovascular-related hospitalizations; (3) all-cause and disease-specific mortality; (4) patient-reported outcome measures (eg, mental well-being, depression, and anxiety questionnaires); and (5) QoL, quality-adjusted life years, and any other health economic outcomes reported in the studies. All the studies that reported one or more of these outcomes were considered eligible for inclusion.

### Search Strategy

The search strategy was developed by the review team, which agreed on the key terms. Medical Subject Headings terms and synonyms for the different terms, such as “telemedicine,” “digital monitoring,” and “e-health” (Table S1 in Multimedia Appendix 1 [13-163]), were used and combined with Boolean operators, proximity operators, truncations, and wildcards. MEDLINE, Scopus, and CENTRAL (the Cochrane Central Register of Controlled Trials) were searched from the date of inception to February 5, 2021, for relevant studies. There were no language restrictions, but the availability of the full text was a requirement for inclusion. Search results were managed using EndNote (version X9.3.3; Clarivate Analytics).

### Study Selection

Two reviewers (MC and DGL) independently screened the titles and abstracts of the studies retrieved from the databases against the search criteria. Additional screening of the preliminary results was independently undertaken by 3 other reviewers (BB, SH, and MI). The full texts of all potentially relevant articles were retrieved and independently assessed by the reviewers in duplicate. Any disagreement was resolved through discussion with the senior author (DL).

### Data Extraction

Data extraction was conducted independently by 2 reviewers (DGL and MC). The following information was extracted: (1) authors, year, country, and reference; (2) study aim; (3) study characteristics (study design and sample size); (4) participant characteristics (age, sex, and ethnicity); (5) health condition; (6) intervention (type of telemedicine device, input of the data [manual or automated], delivery of the intervention, staff involved, duration and frequency of the intervention, and follow-up points); (7) comparators (usual care, different intervention, or no intervention); and (8) outcomes (primary and secondary, as reported in the study).

### Risk of Bias Assessment

Six authors (DGL, MC, BB, SH, MI, and DL) independently assessed the individual studies for risk of bias in duplicate, and any discrepancies were resolved via discussion or referral to a third reviewer, as required. For RCTs, the Cochrane Risk of Bias version 2 tool [164] was used. For nonrandomized studies, the Risk Of Bias In Non-randomized Studies of Interventions [165] was used.

### Data Synthesis

Meta-analyses were conducted on comparable studies. Primary and secondary outcome effect measures with 95% CIs were pooled using the RevMan software (The Cochrane Collaboration) [166]. The results are presented visually using forest plots. Where continuous data were not homogeneous, an estimate of the standardized mean difference (MD) with 95% CIs was calculated. For studies in which quantitative data were too few or too heterogeneous, a narrative synthesis approach was used.

Dichotomous analyses were conducted using the number of events and total sample size as reported in the included studies. The results of the selected studies were combined using the Mantel-Haenszel method. Effect sizes are expressed as relative risk and 95% CIs. Random effect models were applied to all meta-analyses owing to heterogeneity in study characteristics and populations. Heterogeneity was quantitatively assessed using the Higgins index (*I*^2^).

For the analysis of QoL, the postintervention scores, as reported in the included studies, were used. Where the SD was not reported, it was calculated using the calculator function available in RevMan. For analysis of changes in physiological parameters (BP and glycated hemoglobin [HbA_1c_]) and QoL, the results of the selected studies were combined using the generic inverse variance method. Effect sizes are expressed as the MD and SD.

Findings from the included qualitative studies will be synthesized elsewhere using a meta-aggregative approach to data synthesis.

## Results

### Overview

**Figure 1:**
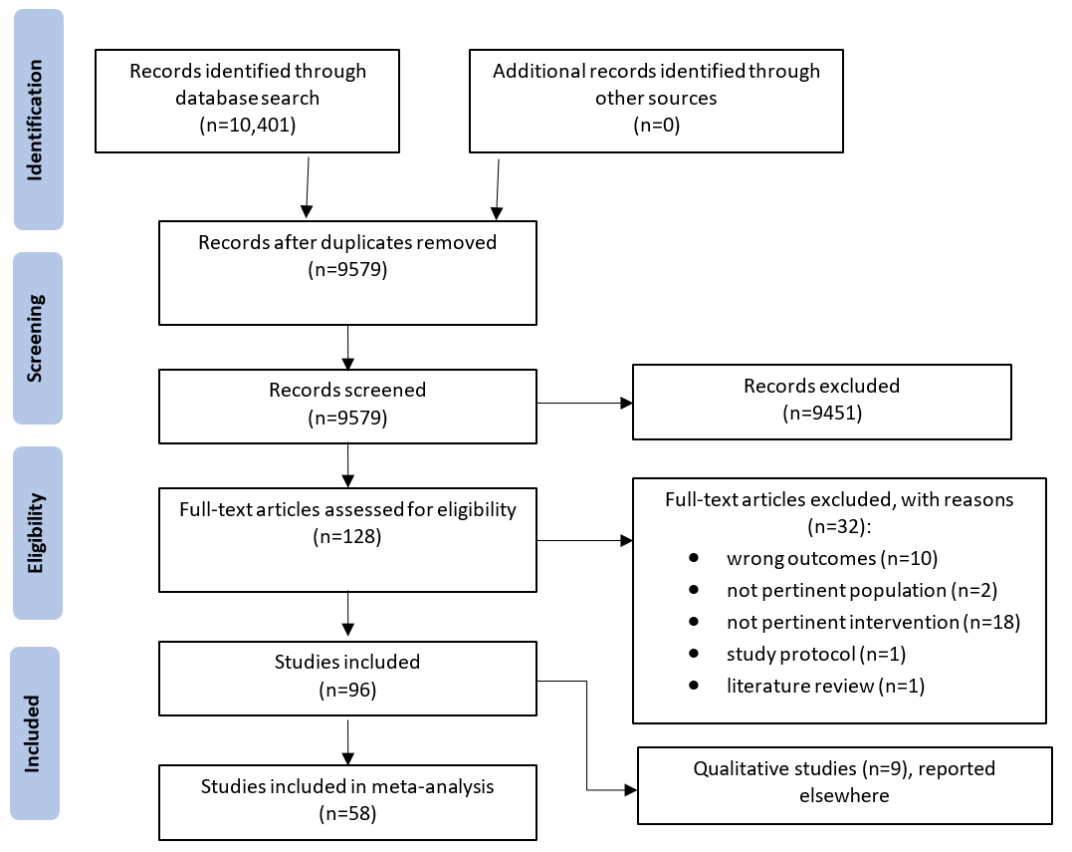
PRISMA (Preferred Reporting Items for Systematic Reviews and Meta-Analyses) diagram depicting the screening and study selection process.

The database searches identified 10,401 papers. After independent screening of titles and abstracts by 2 study authors, 98.77% (10,273/10,401) of papers were determined to be duplicates or not eligible. After screening against the inclusion and exclusion criteria, of the remaining 128 papers, 96 (75%) were included. No ongoing studies were found (Figure 1). A full list of the excluded studies with reasons for exclusion is provided in Table S2 in Multimedia Appendix 1. Full texts of all 96 included papers [13-109] were retrieved.

No study reporting outcomes related to intervention uptake, study retention, and patient acceptability were identified in our search and, therefore, these outcomes could not be analyzed. The following analyses and results concern only patient adherence and satisfaction as well as clinical and patient-reported outcomes.

### Characteristics of the Included Studies

The included studies were published between 1998 and 2020, with sample sizes ranging from 20 [36,99] to 3562 [102] participants and a total sample of 26,167 participants. The mean age ranged from 44 [22] to 78 [107] years, and the proportion of men varied from 25% [51] to 76% [91]. Most of the included studies were conducted in the United Kingdom (21/96, 22%) and the United States (29/96, 30%), with additional studies conducted in Belgium (2/96, 2%), Canada (4/96, 4%), Denmark (5/96, 5%), Poland (2/96, 2%), Singapore (2/96, 2%), South Korea (2/96, 2%), Spain (9/96, 9%), Germany (4/96, 4%), and Italy (6/96, 6%; Multimedia Appendix 2 [13-109,136]). In addition, the following countries had 1% (1/96) of the studies each: Australia [37], China [99], Finland [106], Greece [49], Hong Kong [28], Israel [14], Japan [66], Malaysia [67], the Netherlands [25], and Taiwan [29] (Multimedia Appendix 2).

Populations in the included studies comprised patients with diabetes (27/96, 28% of the studies), cardiovascular disease (stroke, atrial fibrillation, hypertension, and heart failure; 52/96, 54% of the studies), COPD (12/96, 12% of the studies), and mixed chronic conditions (diabetes, hypertension, and COPD; 5/96, 5% of the studies; Multimedia Appendix 2).

### Types of Interventions

The studies varied in their design, type of telemonitoring system used, and method of delivery (Multimedia Appendix 2). Most (64/96, 67%) were RCTs, with 4% (4/96) being nonrandomized controlled studies, 2% (2/96) being cluster randomized studies, 10% (10/96) being longitudinal studies, 4% (4/96) being retrospective analyses, 3% (3/96) being pre-post analyses, and 9% (9/96) having a mixed methods or qualitative design. Most studies (88/96, 92%) used telemonitoring systems that collected patient information via computers, tablets, or dedicated devices (eg, modem) and transferred these data to a web-based server. Some studies collected patient data via SMS text message (3/96, 3%) or by telephone (4/96, 4%). A total of 4% (4/96) of the studies provided educational videos to increase the patients’ knowledge of the disease. The length of the intervention was highly variable, with 5% (5/96) of the studies assessing it over a short period (7-45 days), 21% (20/96) assessing it over a 2- to 4-month period, and most interventions (76/96, 79%) lasting 6 to 12 months. The follow-up periods were inconsistent among the studies and, where present, ranged from 3 to 18 months.

### Types of Comparators

Most studies (79/96, 82%) compared the intervention with usual care, which consisted of routine visits (outpatient clinics) and in-person consultations with general practitioners or the hospital care team (Multimedia Appendix 2). A total of 10% (10/96) of the studies did not have a control group. A total of 1% (1/96) of the studies asked the control group to manually record their data in a diary. In total, 2% (2/96) of the studies used educational videos in the control group to improve patients’ knowledge of the disease, another 2% (2/96) compared the intervention with another telemonitoring device, and 1% (1/96) compared the intervention (telemonitoring device) with telephone communication. A total of 1% (1/96) of the studies used a similar intervention as the control group comparing patients with and without heart failure.

### Types of Outcomes

In total, 12 studies reported adherence to the intervention, including 9 (75%) in patients with cardiovascular disease, 2 (17%) in patients with diabetes, and 1 (8%) in patients with COPD (Multimedia Appendix 2). Patient satisfaction with the intervention was assessed in 9% (9/96) of the studies (2/9, 22% in patients with cardiovascular disease; 3/9, 33% in patients with diabetes; 2/9, 22% in patients with COPD; and 2/9, 22% in a mixed population; Multimedia Appendix 2).

Most studies (31/96, 32%) reported changes in physiological parameters, which varied depending on the population observed, with 39% (12/31) of these studies reporting BP values for patients with cardiovascular disease, 55% (17/31) reporting HbA_1c_ values for patients with diabetes, and 6% (2/31) reporting multiple physiological values in mixed populations (Multimedia Appendix 2).

Hospital admission during the intervention was recorded in 29% (28/96) of the studies (21/28, 75% in patients with cardiovascular disease; 4/28, 14% in patients with COPD; and 3/28, 11% in a mixed sample), and death was noted in 18% (17/96) of the studies (14/17, 82% in patients with cardiovascular disease; 2/17, 12% in patients with COPD; and 1/17, 6% in a mixed population; Multimedia Appendix 2).

QoL before and after the intervention was recorded in 22% (21/96) of the studies (11/21, 52% in patients with cardiovascular disease; 2/21, 10% in patients with diabetes; 6/21, 29% in patients with COPD; and 2/21, 10% in a mixed population; Multimedia Appendix 2).

### Excluded Studies

A total of 25% (32/128) of the studies assessed for eligibility [110-141] were excluded. A summary of these studies can be found in Table S2 in Multimedia Appendix 1. Most (18/32, 56%) were excluded as they were not related to a telemonitoring intervention, 6% (2/32) included disease populations not covered in this review, 31% (10/32) reported outcomes outside the scope of this review, 3% (1/32) were literature reviews, and 3% (1/32) were study protocols.

### Risk of Bias Assessment

A summary of the risk of bias assessment of the included studies can be found in Tables S3-S5 in Multimedia Appendix 1. Overall, most RCTs (48/66, 73%) and non-RCTs (17/20, 85%) included in this review showed either some concerns or a high risk of bias. Most RCT studies (45/66, 68%) showed either some concerns or a high risk of bias in the randomization process as well as in the selection of the reported results. Some RCTs (18/66, 27%) showed either some concerns or a high risk of bias in missing outcome data. Few RCTs (17/66, 26%) showed either some concerns or a high risk of bias in the measurement of the outcomes.

Most of the non-RCTs (18/20, 90%) showed either some concerns or a high risk of bias in the *bias due to confounding* category. A total of 50% (10/20) of the studies showed either some concerns or a high risk of bias in the *bias in measurement of outcomes* category. Few of the non-RCTs (9/20, 45%) showed either some concerns or a high risk of bias in the *bias due to missing data* category as well as in the *bias due to deviations from the intended intervention* category.

The studies included in the meta-analyses were assessed for publication bias. Funnel plots and Egger tests were performed only where ≥10 studies were available [167].

Funnel plots for the outcomes of systolic BP (SBP), HbA_1c_, and mortality can be found in Figures S1-S6 in Multimedia Appendix 1. The Egger test results revealed no evidence of publication bias for SBP, HbA_1c_, or mortality.

### Ongoing Studies

The database search did not return any protocols for ongoing studies. Searches on ClinicalTrials.gov (updated to February 5, 2021) identified 22 ongoing studies [142-163] (n=14, 64% on patients with cardiovascular disease; n=4, 18% on patients with diabetes; and n=4, 18% on patients with COPD), which are reported in detail in Table S6 in Multimedia Appendix 1.

### Primary Outcomes

#### Adherence

Adherence was assessed in 12 studies at different time points: 1 month (n=3, 25%) [51,66,84], 6 weeks (n=2, 17%) [58,103], 2 months (n=1, 8%) [13], 3 months (n=1, 8%) [30], 6 months (n=4, 33%) [42,48,59,92], and 12 months (n=1, 8%) [36]. Of the 12 studies, 7 (58%) [13,36,42,48,58,59,92] demonstrated a benefit of telemonitoring on patient adherence when compared with a comparator, whereas 4 (33%) [30,51,66,84] showed no difference when compared with a comparator. A total of 8% (1/12) of the studies [103] compared 2 telemonitoring systems and showed that educational support combined with telemonitoring positively influenced adherence compared with telemonitoring alone. Owing to variations in how adherence was defined in the studies, a meta-analysis was not performed. A summary of these studies is presented in Table 1.

**Table 1:** Studies examining the impact of telemonitoring interventions versus comparator on adherence (N=12).

Study type and authors, year, and country		Study population, N	Condition		Intervention type, number of participants, age (years), men (n [%])		Comparator, number of participants, age (years), mean (n [%])		Outcomes			Follow-up	Impact of telemonitoring
**Randomized controlled trials**													
	Ong et al [84], 2016, United States	1437	CHF^a^		Automated upload of data on dedicated device or software, 715, mean 73 (SD not reported), men: 382 (53.4); women: 333 (46.6)		Usual care, 722, mean 73 (SD not reported), men: 382 (53.4); women: 333 (46.6)		Adherence electronically recorded; 82.7%			1 month	=^b^
	Gallagher et al [51], 2017, United States	40	HF^c^		Manual upload of data on dedicated device or software, 20, median 68 (IQR 49-79), men: 15 (75); women: 5 (25)		Usual care, 20, median 62 (IQR 52-75), men: 15 (75); women: 5 (25)		Adherence recorded electronically; 81% in both groups			1 month	=
	Kotooka et al [66], 2018, Japan	183	CHF		Automated upload of data on dedicated device or software, 93, mean 67.1 (SD 12.8), men: 51 (56); women: 39 (44)		Usual care, 91, mean 65.4 (SD 15.6), men: 56 (61); women: 35 (39)		Adherence recorded electronically; 90% at 12 months			12 months	=
	Varon et al [103], 2015, United Kingdom	534	HF		Docobo system (telemonitoring only), 135, mean 69.1 (SD 12.6), not reported		Motiva system (telemonitoring+ educational videos), 399, mean 69.1 (SD 12.6), not reported		Adherence assessed by the amount of missing data during the telemonitoring period			6 weeks	−^d^
	Kardas et al [58], 2016, Poland	60	Type 2 diabetes		Automated upload of data on dedicated device or software, 30, mean 59.9 (SD 5.31), men: 17 (57); women: 13 (43)		Usual care, 30, mean 59 (SD 8.9), men: 19 (63); women: 11 (47)		Adherence expressed as medication taken vs medication prescribed; 92.9%			6 weeks	+^e^
	Cho et al [30], 2009, South Korea	69	Type 2 diabetes		Mobile app, 35, mean 51.1 (SD 13.1), 26 men; 74 women^f^		Web-based telemonitoring system, 34, mean 51.1 (SD 13.1), 26 men; 74 women^f^		Adherence, self-reported; >70% in both groups			3 months	=
	Seto et al [92], 2012, Canada	100	CHF		Automated upload of data on dedicated device or software, 50, mean 55.1 (SD 13.7), men: 41 (82); women: 9 (18)		Usual care, 50, mean 52.3 (SD 13.7), men: 38 (76); women: 12 (24)		Adherence registered electronically; 80%			6 months	+
	Evans et al [48], 2016, United States	441	HF and healthy		Disease group: automated upload of data on dedicated device or software, 421, mean 71.8 (SD 8.8), 46 men; 54 women^f^		Healthy group: automated upload of data on dedicated device or software, 20, mean 72.2 (SD 4.3), 50 men; 50 women^f^		Adherence checking the amount of data against the participants’ time spent in the study; between 71% and 81%			6 months	+
**Nonrandomized studies**													
	Agboola et al [13], 2013, United States	30		Hypertension		Web-based device, 15, mean 61.9 (SD not reported), 20 men; 80 women^f^		Mobile blood pressure device, 15, mean 61.6 (SD not reported), 20 men; 80 women^f^		Adherence recorded electronically based on frequency of data transmission	2 months		+
	Domingo et al [42], 2012, Spain	97		HF		Automated upload of data on dedicated device or software, 46, mean 66.5 (SD 11.5), men: 14 (30); women: 32 (70)		Usual care, 51, mean 66.5 (SD 11.5), men: 15 (30); women: 36 (70)		Adherence based on the number of educational videos watched; between 67% and 85%	6 months		+
	Karg et al [59], 2012, Germany	36		COPD^g^		Automated upload of data on dedicated device or software, 36, mean 67.9 (SD 6.9), men: 27 (75); women: 9 (25)		N/A^h^		Adherence: use of the device for at least two-thirds of working days; full compliance	6 months		+
	De Lusignan et al [36], 2001, United Kingdom	20		CHF		Manual upload of data on dedicated device or software, 10, mean 75.2 (SD not reported), not reported		Usual care, 10, mean 75.2 (SD not reported), not reported		Adherence based on the frequency of the uploaded data; 90%	12 months		+

^a^CHF: congestive heart failure.

^b^No differences between telemonitoring and usual care.

^c^HF: heart failure.

^d^Negative impact of telemonitoring over comparator.

^e^Positive impact of telemonitoring over comparator.

^f^Absolute value not reported in the paper.

^g^COPD: chronic obstructive pulmonary disease.

^h^N/A: not applicable.

#### Satisfaction

Patient satisfaction with the intervention was assessed in 9 studies (n=2, 22% in patients with cardiovascular disease; n=3, 33% in patients with diabetes; n=2, 22% in patients with COPD; and n=2, 22% in a mixed population; Table 2). A total of 56% (5/9) of the studies [22,28,42,78,91] demonstrated a benefit of telemonitoring on patient satisfaction when compared with a comparator, whereas 44% (4/9) [30,43,44,95] showed no difference when compared with a comparator. Owing to variations in how satisfaction was defined in the studies, a meta-analysis was not performed. A summary of these studies is provided in Table 2.

**Table 2:** Studies examining the impact of telemonitoring interventions versus comparator on satisfaction (N=9).

Study type and authors, year, and country		Study population, N	Condition	Intervention type, number of participants, age (years), mean (n [%])	Comparator, number of participants, age (years), mean (n [%])	Outcomes	Follow-up	Impact of telemonitoring
**Randomized controlled trials**								
	Bergenstal et al [22], 2005, United States	47	Type 2 diabetes	Automated data transmitted via modem, 24, mean 44 (SD 17), 37 men; 63 women^a^	Data transmitted via telephone, 23, mean 45 (SD 13), 39 men; 61 women^a^	Satisfaction: 5-point questionnaire; 4.30 in the phone group and 4.52 in the modem group	4 weeks	=^b^
	Chau et al [28], 2012, Hong Kong	40	COPD^c^	Manual upload of data on dedicated device or software, 22, mean 73.5 (SD 6), men: 21 (95); women: 1 (5)	Usual care, 18, mean 72.2 (SD 6), men: 18 (100); women: 0 (0)	Satisfaction: 10-item questionnaire based on a 5-point system; 91%	2 months	+^d^
	Edmonds et al [44], 1998, Canada	35	Type 2 diabetes	Mobile phone data transmission, 16, not reported, not reported	Usual care, 19, not reported, not reported	Satisfaction: patient questionnaire	3 months	Further studies required
	Cho et al [30], 2009, South Korea	69	Type 2 diabetes	Mobile app, 35, mean 51.1 (SD 13.1), 26 men; 74 women^a^	Web-based telemonitoring system, 34, mean 51.1 (SD 13.1), 26 men; 74 women^a^	Satisfaction: questionnaire, internet vs phone; 81% vs 79%, respectively	3 months	=
	Sicotte et al [95], 2011, Canada	46	COPD	Manual upload of data on dedicated device or software, 23, mean 73.7 (SD 9.6), men: 13 (56); women: 10 (44)	Usual care, 23, mean 75.4 (SD 9.7), men: 13 (56); women: 10 (44)	Satisfaction: 5-point questionnaire; 4.50 score	3 months	=
	Domingo et al [42], 2012, Spain	97	HF^e^	Automated upload of data on dedicated device or software, 46, mean 66.5 (SD 11.5), men: 14 (30); women: 32 (70)	Usual care, 51, mean 66.5 (SD 11.5), men: 15 (30); women: 36 (70)	Satisfaction: 10-point questionnaire; 8.4 score	6 months	+
**Nonrandomized studies**								
	Schoenfeld et al [91], 2004, United States	59	CHF^f^	Manual upload of data on dedicated device or software, 59, mean 64 (SD 14), men: 45 (76); women: 14 (24)	N/A^g^	Satisfaction: 3-point questionnaire; 98.1% indicating ease of use of the device	7 days	+
	Donate-Martinez et al [43], 2016, Spain	74	Chronic conditions (COPD, type 2 diabetes, and HF)	Manual upload of data on dedicated device or software, 74, mean 67.95 (SD 11.14), men: 49 (66); women: 25 (44)	N/A	Satisfaction: 11-item questionnaire with 10-point score; 8.63 score overall	12 months	=
	Mira-Solves et al [77], 2014, Spain	410	Chronic conditions (type 2 diabetes, hypertension, CHF, and COPD)	Automated upload of data on dedicated device or software, 410, not reported, 64 men; 36 women^a^	N/A	Satisfaction: questionnaire, 89.4% were satisfied with the ease of use.	24 months	+

^a^Absolute value not reported in the paper.

^b^No differences between telemonitoring and usual care.

^c^COPD: chronic obstructive pulmonary disease.

^d^Positive impact of telemonitoring over comparator.

^e^HF: heart failure.

^f^CHF: congestive HF.

^g^N/A: not applicable.

### Secondary Outcomes

#### QoL Measurement

Studies included in the meta-analyses were pooled by comparable scales (eg, the Short Form 36 Health Survey Questionnaire) and end points (eg, 6 or 12 months), with 8% (8/96) of the studies [16,31,33,35,47,96,101,104] included in the meta-analyses.

**Figure 2:**
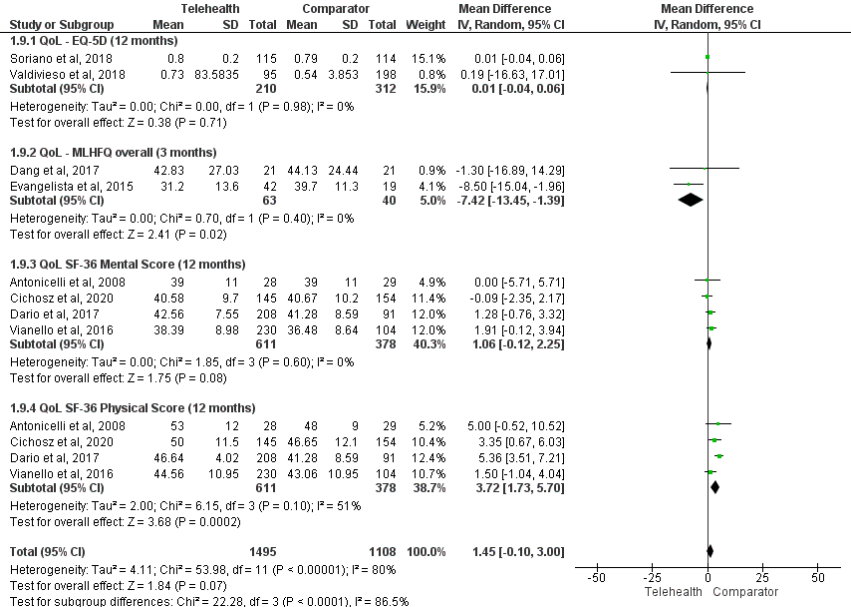
Impact of telemonitoring versus comparator on quality of life (QoL). 1.9.1: EQ-5D; 1.9.2: Minnesota Living with Heart Failure Questionnaire (MLHFQ); 1.9.3: SF-36 mental score; and 1.9.4: SF-36 physical component [15,31,35,47,96,101,104,136].

**Figure 3:**
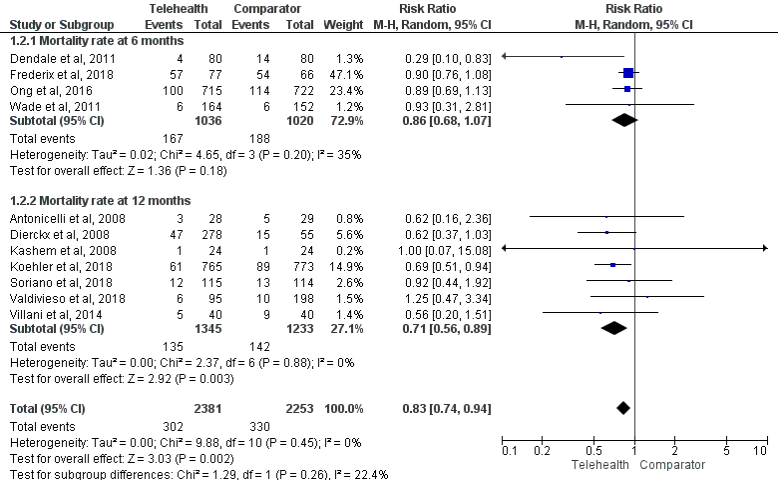
Impact of telemonitoring versus comparator on the mortality rate at 6 and 12 months. The study by Mortara et al [80] was not included in the mortality meta-analyses because of the use of a composite outcome of mortality and hospitalization where absolute mortality results were not available. The study by Seto et al [92] was not included in the mortality meta-analyses because of 0 events in the control group [15,39,40,50,60,64,84,96,101,105,107].

A total of 50% (4/8) of these studies [16,31,35,104] reported the Short Form 36 Health Survey Questionnaire scores (mental and physical) at comparable end points (12 months) and were included in the meta-analyses (Figure 2 [15,31,35,47,96,101,104,136], subgroups 1.9.3 and 1.9.4). From the meta-analysis, telemonitoring showed greater improvements compared with usual care on physical component scores (weighted MD=3.72, 95% CI 1.73-5.70; *P*<.001; *I*^2^=51%; Figure 2) compared with the comparator but no difference in mental component scores (weighted MD=1.06, 95% CI −0.12 to 2.25; *P*=.08; *I*^2^=0%; Figure 3 [15,39,40,50,60,64,84,96,101,105,107]).

In total, 25% (2/8) of the studies [96,101] reported EQ-5D scores at comparable end points (12 months) and were included in the meta-analysis (Figure 2, subgroup 1.9.1). There was no difference in QoL between the groups (weighted MD=0.01, 95% CI −0.04 to 0.06; *P*=.71; *I*^2^=0%)

A total of 25% (2/8) of the studies [33,47] using the Minnesota Living with Heart Failure Questionnaire overall scores at 3 months were included in the meta-analysis (Figure 2, subgroup 1.9.2), demonstrating that the telemonitoring group showed greater improvements in QoL (weighted MD=−7.42, 95% CI −13.45 to −1.39; *P*=.02; *I*^2^=0%) compared with the comparator.

A total of 14% (13/96) of the studies [20,23,36,43,58,62,65,70,92,100,103,107,108] could not be included in the meta-analysis because they reported different time points and used different questionnaires to assess QoL. Of these 13 studies, 4 (31%) reported a significant improvement in QoL in the telemonitoring group compared with usual care at 6 weeks [58], 6 months [92,100], and 12 months [43] measured using a variety of questionnaires (Minnesota Living with Heart Failure Questionnaire [92], EQ-5D [43,58], and 15D [100]), whereas 9 (69%) reported no difference in QoL between telemonitoring and usual care at 4 weeks [70], 6 weeks [65,103], 7 weeks [70], 3 months [36], 6 months [23,62,107], 9 months [108], and 12 months [36]. A total of 8% (1/13) of the studies [20] reported significant improvement in QoL in the usual care group compared with telemonitoring at 2 and 6 months using the St George’s Respiratory Questionnaire.

#### Mortality

Meta-analyses for mortality were conducted at the 6- and 12-month follow-up (Figure 3). Sensitivity analyses were conducted at the 6- and 12-month follow-up excluding studies at high risk of bias and at 12 months excluding non-RCTs (Figure S1 in Multimedia Appendix 1). A sensitivity analysis with the exclusion of non-RCTs at 6 months was not conducted as all the studies included were RCTs.

A total of 11 studies contributed to the all-cause mortality meta-analysis: 4 (36%) [39,50,84,107] (N=2056) provided data at 6 months, and 7 (64%) [16,40,61,64,96,101,105] (N=2578) provided data at 12 months. There was no significant difference in all-cause mortality between telemonitoring and the comparator at 6 months (risk ratio [RR]=0.86, 95% CI 0.68-1.07; *P*=.18; *I*^2^=35%; Figure 3). This finding was consistent when studies evaluated as having a high risk of bias were excluded (Figure S1 in Multimedia Appendix 1). There was a significantly lower risk of all-cause mortality with telemonitoring than with the comparator at 12 months (RR=0.71, 95% CI 0.56-0.89; *P*=.003; *I*^2^=0%; Figure 3). This finding was consistent following the exclusion of non-RCTs and studies evaluated as having a high risk of bias (Figure S1 in Multimedia Appendix 1).

#### Hospitalization

**Figure 4:**
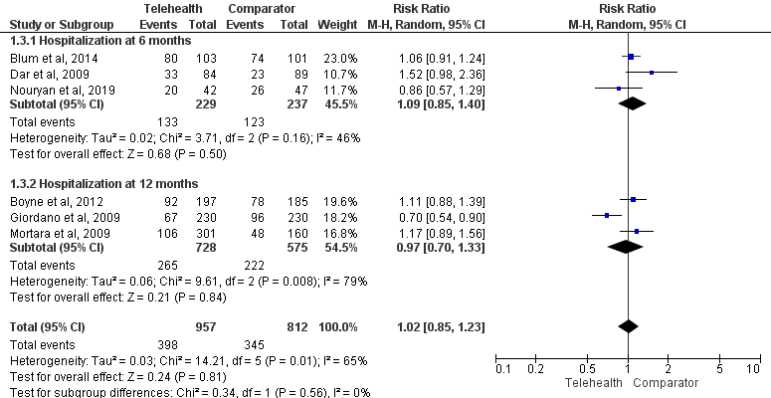
Impact of telemonitoring versus comparator on hospitalization at 6 and 12 months [23,25,34,52,80,83].

Meta-analyses for hospitalization at the 6- and 12-month follow-up were conducted (Figure 4 [23,25,34,52,80,83]), with sensitivity analyses excluding studies classified as having a high risk of bias (Figure S2 in Multimedia Appendix 1) and a subgroup analysis including only studies on patients with heart failure (12/96, 12%). Subgroup analyses for studies on patients with COPD and multiple chronic conditions were not possible because of a lack of absolute values or comparator [29,85].

A total of 8 studies contributed to the all-cause hospitalization meta-analyses: 3 (38%) [23,34,83] (n=466) provided data at 6 months, and 5 (62%) [25,52,80,96,101] (n=1825) provided data at 12 months. There was no significant difference in the risk of all-cause hospitalization between the groups at 6 months (RR=1.09, 95% CI 0.85-1.40; *P*=.50; *I*^2^=46%) or 12 months (RR=0.97, 95% CI 0.70-1.33; *P*=.84; *I*^2^=79%; Figure 4). This result was also consistent after the exclusion of studies evaluated as having a high risk of bias (Figure S2 in Multimedia Appendix 1). The meta-analysis that included only patients with heart failure showed no difference in the risk of hospitalization between the telemonitoring and comparator groups (RR=0.99, 95% CI 0.81-1.22; *P*=.94; *I*^2^=69%; Figure S2 in Multimedia Appendix 1).

#### Changes in BP

A total of 10% (10/96) of the studies [16,17,24,38,45,62,72,75,77] reporting on the change in SBP and 8% (8/96) of the studies [15,17,24,45,62,72,75,77,90] reporting on the change in diastolic BP (DBP) between a telemonitoring intervention and usual care were included in the meta-analyses. Further details on the analyses of BP are provided in Multimedia Appendix 1.

#### Changes in SBP

**Figure 5:**
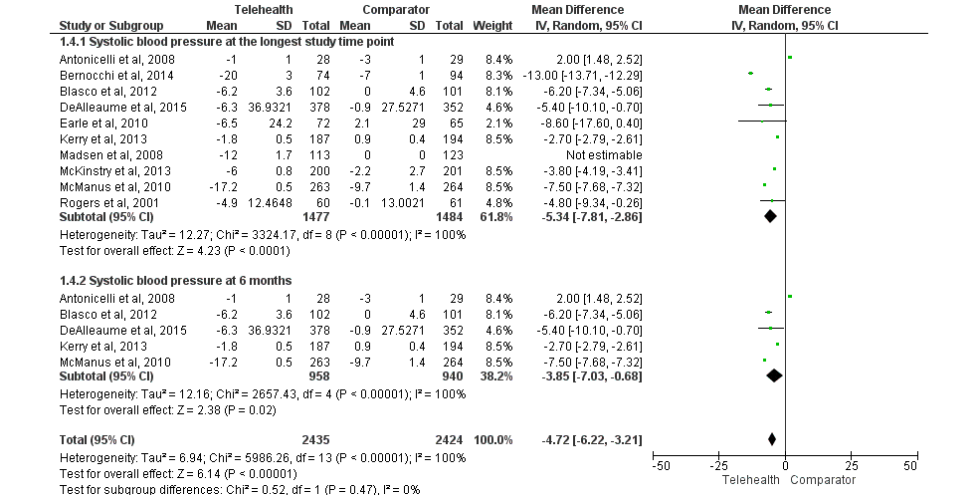
Impact of telemonitoring versus usual care on changes in systolic blood pressure (mean difference) at the longest study time point and at 6 months [15,17,24,38,45,62,72,75,77,90].

SBP was significantly reduced in the telemonitoring group (n=1477) compared with that in the usual care group (n=1484; weighted MD=−5.34 mm Hg, 95% CI −7.81 to −2.86; *P*<.001; *I*^2^=100%; Figure 5 [15,17,24,38,45,62,72,75,77,90]). In the subgroup analysis according to study time points, similar results were observed for SBP at 6 months (weighted MD=−3.85 mm Hg, 95% CI −7.03 to −0.68; *P*=.02; *I*^2^=100%; Figure 5) and 12 months (weighted MD=−3.85 mm Hg, 95% CI −7.03 to −0.68; *P*=.02; *I*^2^=100%; Figure S3 in Multimedia Appendix 1) in favor of telemonitoring.

The sensitivity analysis, excluding studies where the SD was not reported directly [38,45,90], did not materially change the results (weighted MD=−5.19 mm Hg, 95% CI −8.01 to −2.37; *P*<.001; *I*^2^=100%; Figure S3 in Multimedia Appendix 1). The sensitivity analysis was also performed excluding studies with a high risk of bias (Figure S3 in Multimedia Appendix 1); the results remained in favor of telemonitoring (weighted MD=–2.84 mm Hg, 95% CI −4.22 to −1.46; *P*<.001; *I*^2^=98%).

#### Changes in DBP

A meta-analysis including the longest time point demonstrated a significant reduction in DBP in favor of telemonitoring (n=1218) compared with the comparator (n=1255; weighted MD=−2.83 mm Hg, 95% CI −3.98 to −1.68; *P*<.001; *I*^2^=99%; Figure S4 in Multimedia Appendix 1). In the subgroup analysis, a similar result was observed for DBP reduction at 6 months (weighted MD=−5.44 mm Hg, 95% CI −9.00 to −1.87; *P*=.003; *I*^2^=100%; Figure S4 in Multimedia Appendix 1) in favor of telemonitoring but not for DBP at 12 months (weighted MD=−1.09 mm Hg, 95% CI −4.76 to 2.57; *P*=.56; *I*^2^=97%; Figure S4 in Multimedia Appendix 1). Sensitivity analyses at the longest time point excluding studies with high risk of bias (Figure S4 in Multimedia Appendix 1) showed no significant reduction in DBP in the telemonitoring group (weighted MD=−1.07 mm Hg, 95% CI −2.58 to 0.44; *P*=.16; *I*^2^=98%) compared with usual care.

#### Changes in HbA_1c_

A total of 19% (18/96) of the studies reported on HbA_1c_, and all the studies (18/18, 100%) compared telemonitoring with usual care, with 61% (11/18; n=3277) included in the meta-analysis [27,30,35,46,49,58,63,87,89,94,109]. Further details on the excluded studies for the meta-analysis are provided in Multimedia Appendix 1.

The duration of the interval before and after varied, with 18% (2/11) of these studies reporting a 6-week assessment [58,87], 45% (5/11) [27,30,46,49,63] reporting 3-month assessments, 9% (1/11) reporting 9-month assessments [109], and 27% (3/11) [35,89] reporting 12-month assessments. A sensitivity analysis was performed excluding studies with a high risk of bias [58,94].

The overall mean change in HbA_1c_ is shown in Figure S5 in Multimedia Appendix 1. The pooled estimate showed a reduction in the mean change in HbA_1c_ in the telemonitoring group (n=1703; weighted MD=−0.33, 95% CI −0.57 to −0.09; *P*=.008; *I*^2^=99%; Figure S5 in Multimedia Appendix 1). The results did not materially change after the sensitivity analysis excluding studies at high risk of bias [58,87] (Figure S5 in Multimedia Appendix 1). Subgroup analyses according to study time points showed no significant difference in the change in HbA_1c_ values between telemonitoring and the comparator (Figure S5 in Multimedia Appendix 1).

## Discussion

### Principal Findings

Our results suggest that telemonitoring interventions are associated with good patient adherence and satisfaction. Although this review did not demonstrate improvements in QoL with telemonitoring, there was evidence to suggest reductions in all-cause mortality and improvements in BP and blood glucose control. Conversely, there was evidence to suggest that telemonitoring interventions may be associated with a higher rate of hospitalizations, which could be interpreted as a positive role of telemonitoring in detecting patients’ health issues more than usual care.

### Comparison With Prior Work

Our review showed improvements in physiological parameters (BP and blood glucose) in patients receiving telemonitoring interventions. These findings demonstrate the positive role of telemonitoring in improving patients’ self-management of their conditions. This is in line with other reviews that have shown similar improvements in hypertension [168] and type 2 diabetes self-management [169] after telemonitoring interventions.

The studies included in this review consistently showed that patients receiving telemonitoring interventions had lower all-cause mortality compared with patients receiving usual care. A recent umbrella review [170] examining the effects of telemonitoring on mortality in several clinical populations (cardiovascular, COPD, and neurological) reported similar findings for the cardiovascular population, where the mortality rate was either reduced in the telemedicine users or remained unchanged compared with usual care. The same review [170] did not find any difference in mortality between telemonitoring and usual care in patients with COPD. The impact on death is an important outcome when considering the administration of remote interventions over in-person visits, and the reduced mortality rate with telemonitoring reported in our review suggests the effectiveness of telemonitoring for patients with chronic conditions.

Surprisingly, the overall results of our review showed a higher risk of hospitalization among patients undergoing telemonitoring interventions. There is inconsistency in the previous literature on the role that telemonitoring plays in reducing the risk of rehospitalization, with some studies reporting no differences compared with usual care [171] and others concluding that telemonitoring is an effective tool to reduce all-cause hospitalization in adults with heart failure [172]. Thurmond et al [173] noted the importance that the type of telemonitoring intervention has on its acceptability by patients and, consequently, their adherence to it, which, when poor, may influence the rate of rehospitalization. This would suggest the need to identify common characteristics of effective telemonitoring interventions (or “active ingredients”) that facilitate patient acceptability. It may also be possible that increased hospitalizations with telemonitoring is a positive finding (ie, reasons for hospitalization may be identified earlier by telemonitoring, and hospitalization may be initiated earlier than with usual care, averting serious outcomes and death). Hypothetically, this could have contributed to the reduced mortality at 12 months; however, future research is needed to substantiate this.

The results of this review are in line with those of previous systematic reviews assessing patient satisfaction with telemonitoring interventions [174,175]. From qualitative reports, the convenience of decreased travel time and costs and the reassurance of being monitored are the most likely reasons for patients preferring telemonitoring over usual care [176]. It is important to note that patient satisfaction may differ with the type of telemonitoring device used; indeed, available evidence suggests that higher patient satisfaction is reported for videoconferences and devices that allow for automated data transmission [174].

The included studies did not report significant improvements in the QoL of patients receiving a telemonitoring intervention compared with usual care. Our findings confirm previous reviews [177,178] while expanding the results to populations outside care homes [178] and including study designs other than RCTs [177]. Although telemonitoring does not seem to improve QoL compared with usual care, previous findings [178] have shown important benefits of telemonitoring in improving patients’ confidence in accessing health care services.

### Strengths and Limitations

This review used a strict definition of telemonitoring, only including studies that used a device to collect health measures and facilitated 2-way communication or action between the patient and health care team. Despite the inclusion of studies with low methodological quality, sensitivity analyses were conducted where appropriate, reducing the potential for bias to affect the results of this review. The studies included in this review presented a wide range of telemonitoring interventions that differed in the personnel involved, administration of the intervention, and technology used and that were examined in a variety of populations with different long-term conditions, thus making the results highly generalizable. A robust methodology was used, with independent screening and data extraction by 2 reviewers and risk of bias assessment in duplicate.

Several limitations are noteworthy. First, despite our initial plans to investigate uptake, patient retention and satisfaction, and associated factors when using 2-way (patient-health care provider) remote patient monitoring devices to manage chronic health conditions, no studies reported uptake and retention outcomes and, therefore, these outcomes could not be reported in this review. Most of the included studies assessed similar outcomes but used different measurement tools, thus making comparison difficult, particularly in studies investigating patient adherence [13,30,36,42,48,51,58,59,66,84,92,103] and satisfaction [22,28,30,42-44,78,91,95] with the intervention. Second, despite our efforts to define the best search strategy to identify all relevant articles for our review, the possible omission of papers because of the heterogeneity in the key terms used by the authors cannot be ruled out. We did not conduct any searches for gray literature. Third, most outcomes analyzed in this review have been infrequently investigated in the literature (eg, mortality was reported only in 17/96, 18% of the included studies; adherence was reported in only 12/96, 12% of the studies; and satisfaction was reported in only 9/96, 9% of the studies), and further research is required to properly assess the effects of telemonitoring on these outcomes. Moreover, some conditions (eg, COPD) were underrepresented as few studies investigating the effects of telemonitoring interventions on these populations were available; thus, we could not conduct a separate meta-analysis for each condition. The type and quality of usual care also varied throughout the included studies, which may have influenced the results in favor of or against telemonitoring.

### Conclusions

Telemonitoring is a promising tool to manage long-term conditions, with the potential to reduce the associated costs and alleviate patient difficulties in accessing primary health care. Patient satisfaction and adherence to telemonitoring appear, overall, to be promising. Although telemonitoring resulted in improvement in physiological parameters and reduced all-cause mortality compared with usual care, there was no improvement in QoL and an increased risk of hospitalization with telemonitoring. Although the latter may be a positive finding indicating earlier detection of health issues and action (resulting in hospitalization), this result warrants further investigation. Telemonitoring is expanding rapidly, more so since the COVID-19 pandemic, and has been shown to be a viable alternative to usual care for the management of patients with long-term health conditions.

## Appendix

Multimedia Appendix 1 Supplementary figures and tables that were not included in the main manuscript.

Multimedia Appendix 2 Summary of the included studies (N=96).

## Abbreviations

BP: blood pressure
COPD: chronic obstructive pulmonary disease
DBP: diastolic blood pressure
HbA_1c_: glycated hemoglobin
MD: mean difference
PRISMA: Preferred Reporting Items for Systematic Reviews and Meta-Analyses
PROSPERO: International Prospective Register of Systematic Reviews
QoL: quality of life
RCT: randomized controlled trial
RR: risk ratio
SBP: systolic blood pressure
