# Supplementary material for: Interactive Remote Patient Monitoring Devices for Managing Chronic Health Conditions: Systematic Review and Meta-analysis
Source: J Med Internet Res. 2022 Nov 3;24(11):e35508. doi: 10.2196/35508 (PMC9673001; doi:10.2196/35508)
Supplement: Multimedia Appendix 1 [file jmir_v24i11e35508_app1.doc]

**SUPPLEMENTARY MATERIAL**

Supplementary Table 1: Search Strategy for Ovid MEDLINE

| **Terms** | **Results** |
| --- | --- |
| #1.exp Telemedicine/ | 32,271 |
| #2.exp Telecommunications/ | 97,220 |
| #3.Telecare.mp. | 788 |
| #4.Telehealth.mp. | 6,616 |
| #5.e-technology.mp. | 64 |
| #6.mobile health.mp. | 8,718 |
| #7.interactive.mp. | 56,578 |
| #.8 remote monitoring.mp. | 2,518 |
| #.9 automated alert.mp. | 63 |
| #10.exp Videoconferencing/ | 2,123 |
| #11.digital.mp. | 144,370 |
| #12.exp Self-Management/ | 2,781 |
| #13.exp Telephone/ | 23,302 |
| #14.exp Smartphone/ | 5,212 |
| #15.exp Cell Phone/ | 11.244 |
| #16.anroid.mp. | 2,798 |
| #17.iphone.mp. | 910 |
| #18.IOS.mp. | 1,727 |
| #19.device.mp. | 292,723 |
| #20.mobile device.mp. | 1,292 |
| #21.store-and-forward.mp. | 584 |
| #22.e-health.mp. | 3,067 |
| #23.m-health.mp. | 629 |
| #24.exp Cardiovascular Disease/ | 2,430,366 |
| #25.exp Heart Diseases/ | 1,148,108 |
| #26.exp Heart Failure/ | 125,042 |
| #27.cardiac surgery.mp. | 43,106 |
| #28.exp Thoracic Surgery/ | 12,822 |
| #29.exp Myocardial Infarction/ | 178,034 |
| #30.exp Myocardial Ischemia/ | 436,437 |
| #31.silent myocardial infarction.mp. | 267 |
| #32.silent myocardial ischemia.mp. | 1,162 |
| #33. Exp Coronary Artery Disease/ | 64,329 |
| #34. Exp Angina pectoris/ | 43,665 |
| #35. Exp Stroke/ | 139,798 |
| #36. Exp Pacemaker, Artificial/ | 27,673 |
| #37. Exp Defibrillators/ | 18,722 |
| #38. Exp Defibrillators. Implantable/ or ICD.mp | 47,712 |
| #39. Exp Atrial Fibrillation/ or AF.mp. | 93,862 |
| #40. Afib.mp. | 350 |
| #41. copd.mp. or exp Pulmonary Disease, Chronic Obstructive/ | 75,878 |
| #42.exp Diabetes Mellitus/ | 435,708 |
| #43. Chronic disease.mp or exp Chronic Disease/ | 294,090 |
| #44.exp Hypertension/ | 258,047 |
| #45. Exp Arrhythmias, Cardiac/ | 213,352 |
| #46. #24 or #25 or #26 or #27 or #28 or #29 or #30 or #31 or #32 or #33 or #34 or #35 or #36 or #37 or #38 or #39 or #40 or #41 or #42 or #43 or #44 or #45 | 3,148,500 |
| #47. #1 or #2 or #3 or #4 or #5 or #6 or #7 or #8 or #9 or #10 or #11 or #12 or #13 or #14 or #15 or #16 or #17 or #18 or #19 or #20 or #21 or #22 or #23 | 589,041 |
| #48. #46 and #47 | 88,113 |
| #49 limit #48 to (humans) | 48.017 |
| #50 (randomized controlled trials or controlled clinical trial or pragmatic clinical trial or multicentre study).pt or non-randomized controlled trials as topic/ or interrupted time series analysis/ or controlled before-after- studies/ or (ramdomis* or randomiz* or randomly).ti,ab. or trial.ti. or (before adj5 after).mp. or (pre adj5 post).mp. or ((pretest or pre test) and (posttest or post test)).mp. or quasiexperiment*.mp. or quasi experiment*.mp or time series.mp. or repeated measure*.ti.,ab. | 1,875,395 |
| #51. #49 and #50 | 10,401 |

**Supplementary Table 2 – List of Excluded Studies with Reasons**

| **Study ID** | **Reason(s) for exclusion** |
| --- | --- |
| Aberger, 2014 | Population not pertinent to the review |
| Abraham, 2011 | Intervention not pertinent to the review |
| Abraham, 2013 | Intervention not pertinent to the review |
| Abraham, 2017 | Intervention not pertinent to the review |
| Adamson, 2014 | Intervention not pertinent to the review |
| Adamson, 2016 | Intervention not pertinent to the review |
| Ades, 2000 | Intervention not pertinent to the review |
| Ahring, 1992 | Population not pertinent to the review |
| Ajay, 2016 | Intervention not pertinent to the review |
| Al-Khatib, 2010 | Intervention not pertinent to the review |
| Amara, 2015 | Intervention not pertinent to the review |
| Ando, 2011 | Intervention not pertinent to the review |
| Bastyr, 2015 | Intervention not pertinent to the review |
| Batalik, 2020 | Intervention not pertinent to the review |
| Bekelman, 2015 | Intervention not pertinent to the review |
| Benhamou, 2007 | Intervention not pertinent to the review |
| Bernocchi, 2018 | Wrong Outcomes |
| Bhavnani, 2018 | Intervention not pertinent to the review |
| Biermann, 2000 | Wrong Outcomes |
| Billiard, 1991 | Wrong Outcomes |
| Bohm, 2016 | Intervention not pertinent to the review |
| Bosworth, 2011 | Intervention not pertinent to the review |
| Boyne, 2014 | Wrong Outcomes |
| Bray, 2015 | Wrong Outcomes |
| Chan, 2005 | Intervention not pertinent to the review |
| Chase, 2003 | Wrong Outcomes |
| Dang, 2017 | Wrong Outcomes |
| Desai, 2012 | Literature review |
| Ding, 2017 | Study protocol |
| Schwarz, 2008 | Wrong Outcomes |
| Trief, 2009 | Wrong Outcomes |
| Zakeri, 2020 | Wrong Outcomes |

**Supplementary Table 3 – Risk of Bias assessment for included studies (ITT**) undertaken using the risk of bias- 2 (RoB 2) tool (Sterne et al., 2019)**

| **Study ID: Author, year** | **Randomization Process** | **Deviations from the intended intervention** | **Missing Outcome data** | | **Measurement of the outcome** | | **Selection of the reported result** | | **Overall** | |  |
| --- | --- | --- | --- | --- | --- | --- | --- | --- | --- | --- | --- |
| Antonicelli 2008 |  |  | |  | |  | |  | |  | |
| Baron 2017 |  |  | |  | |  | |  | |  | |
| Bentley 2014 |  |  | |  | |  | |  | |  | |
| Beran 2018 |  |  | |  | |  | |  | |  | |
| Bergenstal 2005 |  |  | |  | |  | |  | |  | |
| Blasco 2012 |  |  | |  | |  | |  | |  | |
| Blum 2014 |  |  | |  | |  | |  | |  | |
| Boyne 2012 |  |  | |  | |  | |  | |  | |
| Chau 2012 |  |  | |  | |  | |  | |  | |
| Cho 2009 |  |  | |  | |  | |  | |  | |
| Cichosz 2020 |  |  | |  | |  | |  | |  | |
| Cleland 2015 |  |  | |  | |  | |  | |  | |
| Dang 2017 |  |  | |  | |  | |  | |  | |
| Dar 2009 |  |  | |  | |  | |  | |  | |
| Dario 2017 |  |  | |  | |  | |  | |  | |
| de Lusignan 2001 |  |  | |  | |  | |  | |  | |
| De San Miguel |  |  | |  | |  | |  | |  | |
| Dendale 2012 |  |  | |  | |  | |  | |  | |
| Dinesen 2012 |  |  | |  | |  | |  | |  | |
| Earle 2010 |  |  | |  | |  | |  | |  | |
| Edmonds 1998 |  |  | |  | |  | |  | |  | |
| Frederix 2019 |  |  | |  | |  | |  | |  | |
| Gallagher 2017 |  |  | |  | |  | |  | |  | |
| Giordano 2009 |  |  | |  | |  | |  | |  | |
| Greenwood 2015 |  |  | |  | |  | |  | |  | |
| Istephanian 2009 |  |  | |  | |  | |  | |  | |
| Kashem 2008 |  |  | |  | |  | |  | |  | |
| Kerry 2013 |  |  | |  | |  | |  | |  | |
| Kim 2008 |  |  | |  | |  | |  | |  | |
| Koehler 2018 |  |  | |  | |  | |  | |  | |
| Kootoka 2018 |  |  | |  | |  | |  | |  | |
| Leng Chow 2020 |  |  | |  | |  | |  | |  | |
| Madigan 2013 |  |  | |  | |  | |  | |  | |
| Margolis 2013 |  |  | |  | |  | |  | |  | |
| McKinstry 2013 |  |  | |  | |  | |  | |  | |
| Mortara 2009 |  |  | |  | |  | |  | |  | |
| Neumann 2011 |  |  | |  | |  | |  | |  | |
| Nouryan 2019 |  |  | |  | |  | |  | |  | |
| Ong 2016 |  |  | |  | |  | |  | |  | |
| Rodriguez-Idigoras 2009 |  |  | |  | |  | |  | |  | |
| Shea 2006 |  |  | |  | |  | |  | |  | |
| Shea 2009 |  |  | |  | |  | |  | |  | |
| Soriano 2018 |  |  | |  | |  | |  | |  | |
| Tupper 2018 |  |  | |  | |  | |  | |  | |
| Valdivieso 2018 |  |  | |  | |  | |  | |  | |
| Vuorinen 2014 |  |  | |  | |  | |  | |  | |
| Wade 2011 |  |  | |  | |  | |  | |  | |
| Walker 2018 |  |  | |  | |  | |  | |  | |
| Weintraub 2010 |  |  | |  | |  | |  | |  | |
| Weintraub 2010 |  |  | |  | |  | |  | |  | |
| Wild 2016 |  |  | |  | |  | |  | |  | |

*green= low risk of bias; yellow= some concerns, red= high risk of bias

**ITT: Intention to Treat

*Sterne JAC, Savović J, Page MJ, Elbers RG, Blencowe NS, Boutron I, Cates CJ, Cheng H-Y, Corbett MS, Eldridge SM, Hernán MA, Hopewell S, Hróbjartsson A, Junqueira DR, Jüni P, Kirkham JJ, Lasserson T, Li T, McAleenan A, Reeves BC, Shepperd S, Shrier I, Stewart LA, Tilling K, White IR, Whiting PF, Higgins JPT. RoB 2: a revised tool for assessing risk of bias in randomised trials. BMJ 2019; 366: l4898.*

**Supplementary Table 4 – Risk of Bias assessment for included studies (PP) undertaken using the risk of bias- 2 (RoB 2) tool (Sterne et al., 2019)**

| **Study ID: Author, year** | **Randomisation Process** | **Deviations from the intended intervention** | **Missing Outcome data** | **Measurement of the outcome** | **Selection of the reported result** | **Overall** |
| --- | --- | --- | --- | --- | --- | --- |
| Domingo 2012 |  |  |  |  |  |  |
| Egede 2017 |  |  |  |  |  |  |
| Fountoulakis 2015 |  |  |  |  |  |  |
| Kardas 2016 |  |  |  |  |  |  |
| Kashem 2006 |  |  |  |  |  |  |
| Konstam 2011 |  |  |  |  |  |  |
| Lewis 2010 |  |  |  |  |  |  |
| McManus 2010 |  |  |  |  |  |  |
| Pressman 2013 |  |  |  |  |  |  |
| Ralston 2014 |  |  |  |  |  |  |
| Seto 2012 |  |  |  |  |  |  |
| Varon 2015 |  |  |  |  |  |  |
| Vianello 2016 |  |  |  |  |  |  |
| Villani 2014 |  |  |  |  |  |  |

*green= low risk of bias, yellow= some concerns, red= high risk of bias

**PP: Per Protocol

*Sterne JAC, Savović J, Page MJ, Elbers RG, Blencowe NS, Boutron I, Cates CJ, Cheng H-Y, Corbett MS, Eldridge SM, Hernán MA, Hopewell S, Hróbjartsson A, Junqueira DR, Jüni P, Kirkham JJ, Lasserson T, Li T, McAleenan A, Reeves BC, Shepperd S, Shrier I, Stewart LA, Tilling K, White IR, Whiting PF, Higgins JPT. RoB 2: a revised tool for assessing risk of bias in randomised trials. BMJ 2019; 366: l4898.*

**Supplementary Table 5 – Risk of Bias assessment for included studies undertaken using the Risk of Bias in non-Randomized Studies – of Interventions (ROBINS-I)** (Sterne et al., 2016)

| **Study ID: Author, year** | **Bias due to confounding** | **Bias in selection of participants into the study** | **Bias in classification of interventions** | **Bias due to deviations from the intended intervention** | **Bias due to missing data** | **Bias in measurement of outcomes** | **Bias in selection of the reported results** | **Overall** |
| --- | --- | --- | --- | --- | --- | --- | --- | --- |
| Agboola 2013 |  |  |  |  |  |  |  |  |
| Amir 2017 |  |  |  |  |  |  |  |  |
| Bernocchi 2014 |  |  |  |  |  |  |  |  |
| Buis 2020 |  |  |  |  |  |  |  |  |
| Chau 2012 |  |  |  |  |  |  |  |  |
| Chen 2013 |  |  |  |  |  |  |  |  |
| DeAlleaume 2015 |  |  |  |  |  |  |  |  |
| Dierckx 2015 |  |  |  |  |  |  |  |  |
| Donate-Martinez 2016 | **NI** |  |  |  | **NI** |  |  |  |
| Evangelista 2015 |  |  |  |  |  |  |  |  |
| Grady 2016 |  |  |  | **NI** |  |  |  |  |
| Karg 2012 |  |  |  |  |  |  |  |  |
| Kim 2008 |  |  |  |  |  |  |  |  |
| Leng Chow 2020 |  |  |  |  |  |  |  |  |
| Michaud 2018 |  |  |  | **NI** |  |  |  |  |
| Mira-Solves 2014 | **NI** |  |  |  |  |  |  |  |
| Orozco-Beltran 2017 |  |  |  |  |  |  |  |  |
| Rodriguez-Idigoras 2009 |  |  |  |  |  |  |  |  |
| Schoenfeld 2004 |  |  |  |  |  |  |  |  |
| Sicotte 2011 | **NI** |  |  | **NI** |  |  |  |  |
| Stuckey 2011 |  |  |  | **NI** |  |  |  |  |
| Trudel 2017 |  | **NI** | **NI** | **NI** | **NI** | **NI** | **NI** | **NI** |
| van Berkel 2019 |  |  |  |  |  |  |  |  |

*green=low risk, yellow= moderate risk, red= severe risk, NI= No information

*Sterne JAC, Hernán MA, Reeves BC, Savović J, Berkman ND, Viswanathan M, Henry D, Altman DG, Ansari MT, Boutron I, Carpenter JR, Chan AW, Churchill R, Deeks JJ, Hróbjartsson A, Kirkham J, Jüni P, Loke YK, Pigott TD, Ramsay CR, Regidor D, Rothstein HR, Sandhu L, Santaguida PL, Schünemann HJ, Shea B, Shrier I, Tugwell P, Turner L, Valentine JC, Waddington H, Waters E, Wells GA, Whiting PF, Higgins JPT. ROBINS-I: a tool for assessing risk of bias in non-randomized studies of interventions. BMJ 2016; 355; i4919; doi: 10.1136/bmj.i4919.*


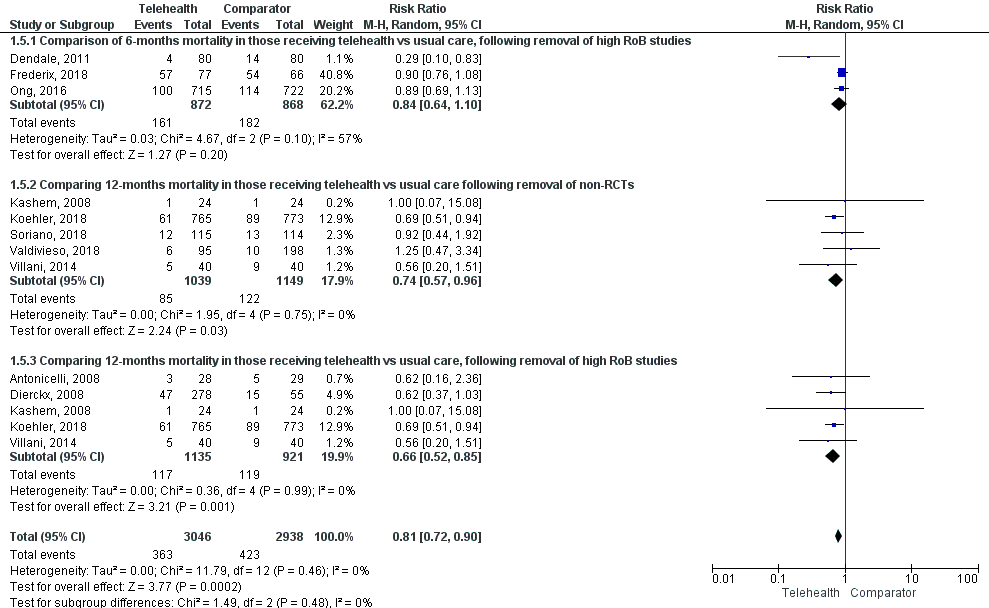


**Supplementary Figure 1:** Impact of telehealth versus comparator on the mortality rate following removal of high risk of bias study at 6 months (1.5.1) and at 12 months (1.5.3); and following removal of non-RCTs at 12 months (1.5.2).


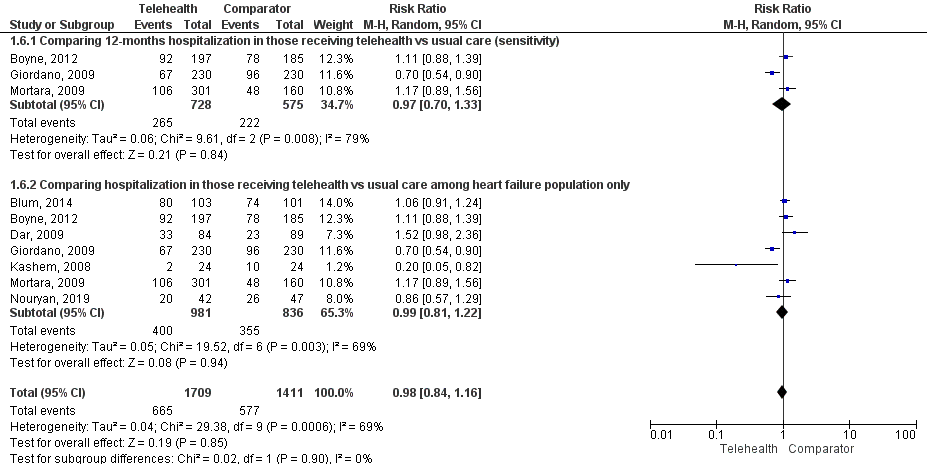


**Supplementary Figure 2:** Impact of telehealth versus comparator on the hospitalization rate following removal of high risk of bias study at 12 months (1.6.1); and among heart failure population only (1.6.2).

**Supplementary File 1. Secondary Outcomes: Changes in Blood Pressure**

Only Rogers et al. [90] reported mean changes for blood pressure (BP). For the remaining studies, mean changes for systolic BP (SBP) [15, 17, 24, 38, 45, 62, 72, 75, 77] and diastolic BP (DBP) [15, 17, 45, 62, 72, 75, 77] were calculated by subtracting the value at baseline from the post-intervention value. For two studies [85, 88] the outcomes were reported as the percentage of people reaching the target BP values, and these studies were excluded from the meta-analysis. Where the SD was not reported by the authors this was estimated from the 95% CI or p-values using RevMan (5.4.1) tools. The SD for deAlleaume et al. [38] were calculated using the p-values for the intervention and comparator, and for two other studies [45, 90] using the 95% CI, and were excluded in sensitivity analyses. Further, sensitivity analysis was performed excluding studies with high risk of bias [17, 38, 77]. For the main analyses, data relating to the longest duration of treatment were considered. Results are also presented based on time-points. Only two studies [62, 77] included multiple time points at 6-months and 12-months.


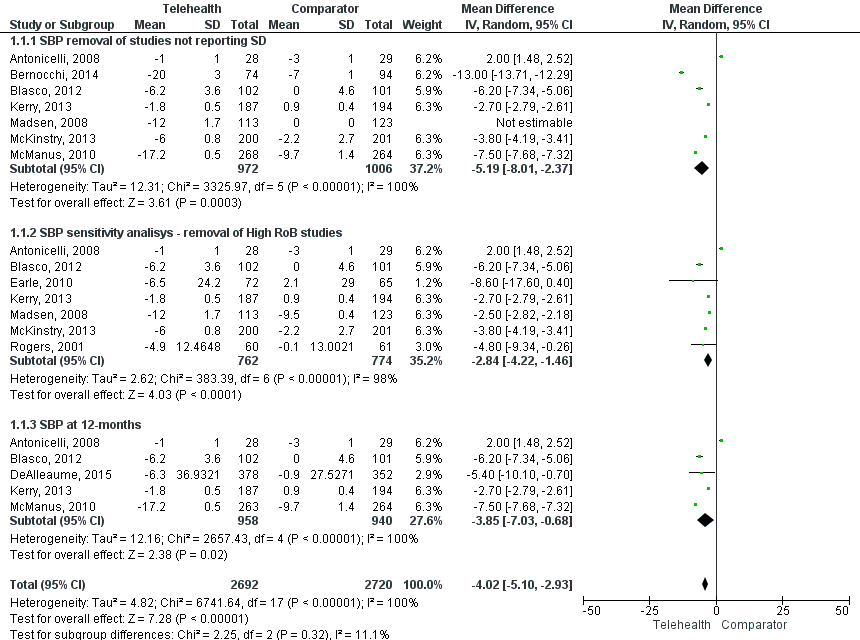


**Supplementary Figure 3:** Impact of telehealth versus usual care on changes in systolic blood pressure (Mean Difference) at 12 months (1.1.3); after removal of studies that did not report SD (1.1.1.); and after removal of high risk of bias studies (1.1.2).


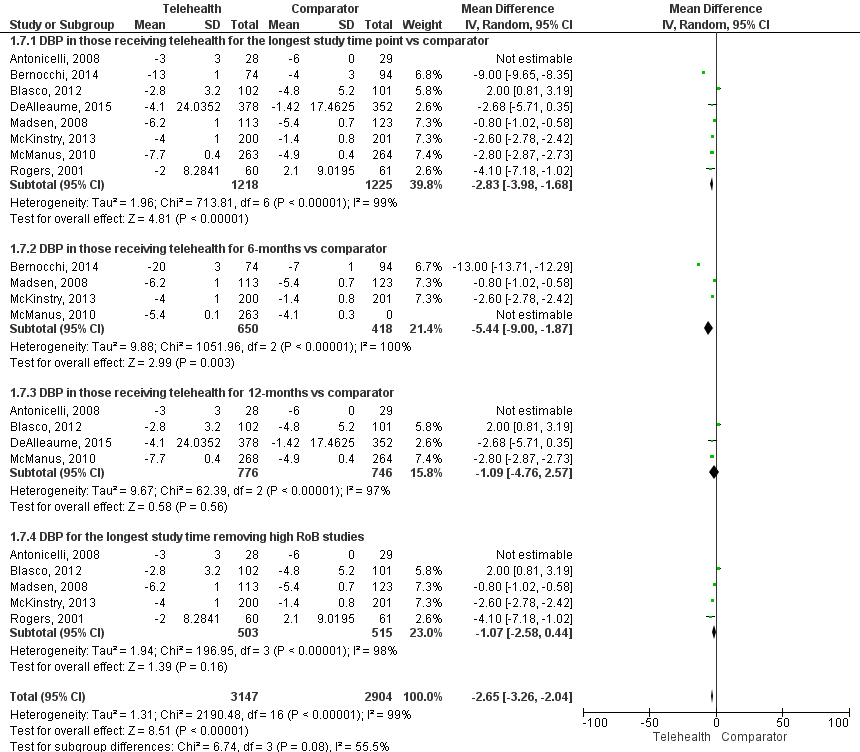


**Supplementary Figure 4:** Impact of telehealth versus usual care on changes in diastolic blood pressure (Mean Difference) at 6 months (1.7.2); at 12 months (1.7.3); and at the longest study time removing high risk of bias studies (1.7.4).

**Supplementary File 2. Secondary Outcomes: HbA1c**

Eighteen studies reported on HbA1c. Seven studies [22, 53, 55, 57, 76, 85, 99] were excluded from the meta-analyses; two [53, 85] were single arm studies, four [22, 55, 57, 76] had insufficient data for calculation of SD, and one [85] reported a mean difference for treatment of 0.0 without corresponding SDs and therefore the result could not be estimated by RevMan. Eleven studies (n=3,277) were included in the meta-analysis [27, 30, 35, 46, 49, 58, 63, 87, 89, 93, 109], of which two [35, 87] reported the mean difference; for the remaining nine studies [27, 30, 46, 49, 58, 63, 89, 93, 109] mean difference was calculated.


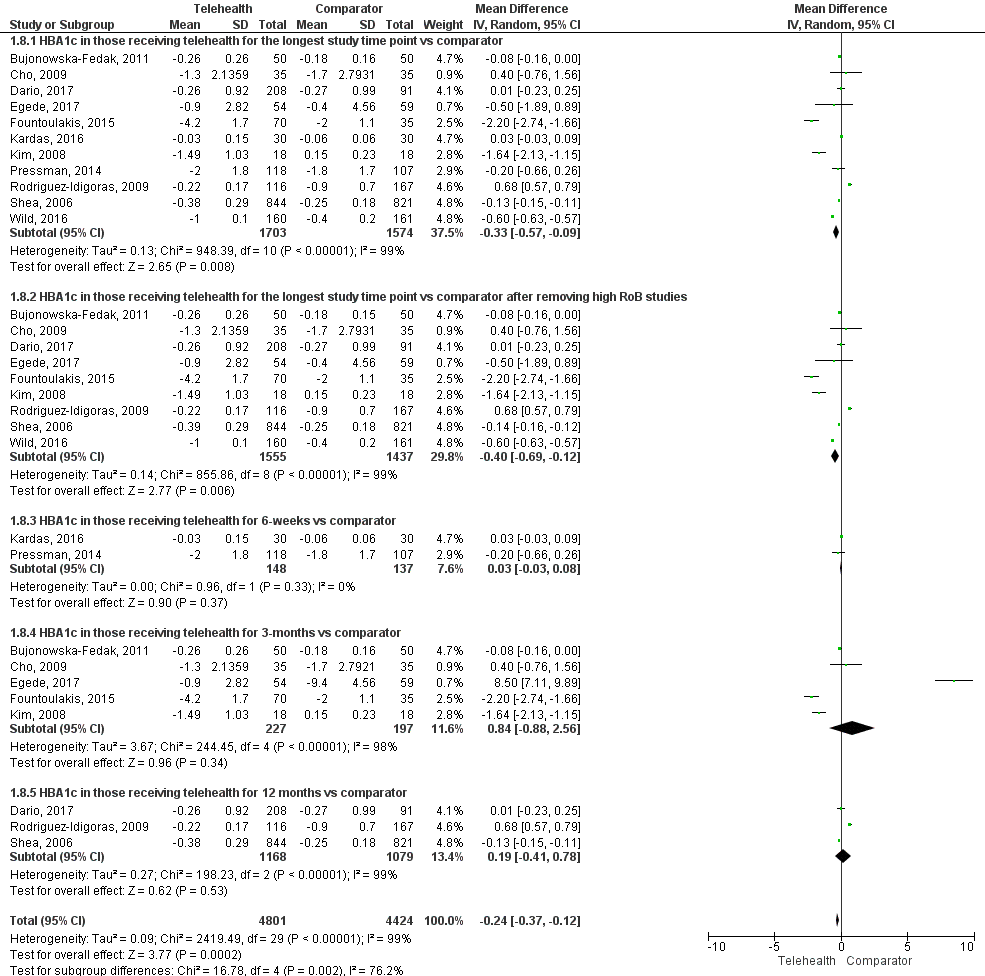


**Supplementary Figure 5:** Impact of telehealth versus usual care on changes in HbA1c (Mean Difference) at the longest study time (1.8.1); after removal of high risk of bias studies (1.8.2); at 6-week time (1.8.3); at 3-month time (1.8.4); and at 12-month time (1.8.5).

**
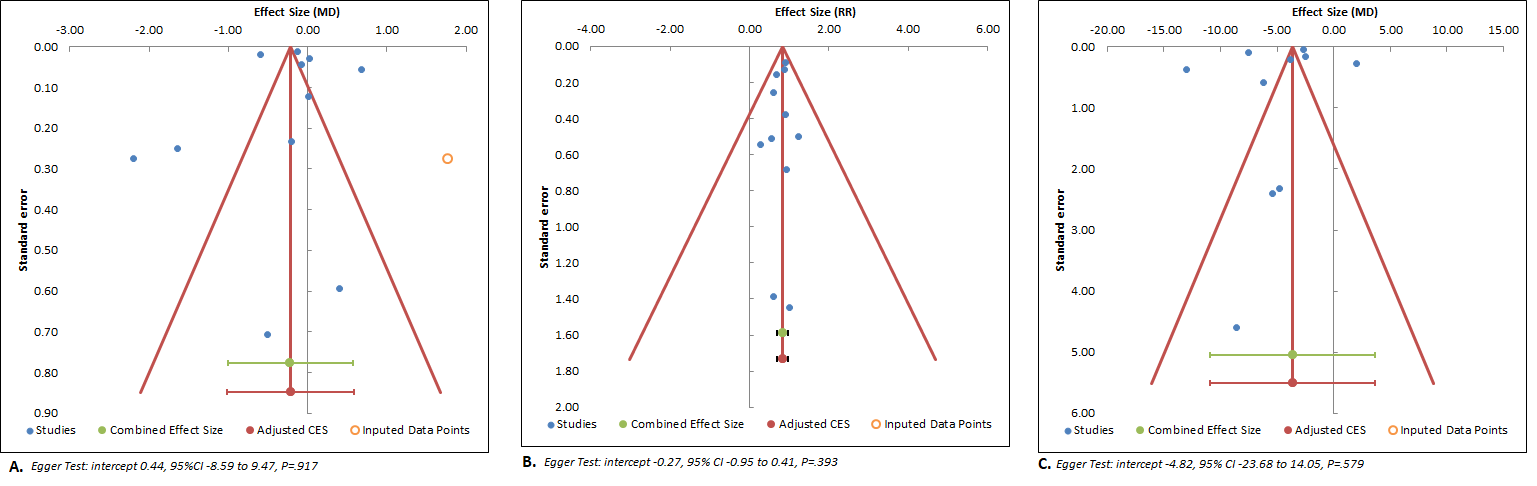
**

**Supplementary Figure 6:** Funnel Plots – A) HbA1c; B) Mortality; C) Systolic Blood Pressure.

Supplementary Table 6 – PRISMA checklist.

| **Section and Topic** | **Item #** | **Checklist item** | **Location where item is reported** |
| --- | --- | --- | --- |
| **TITLE** | | |  |
| Title | 1 | Identify the report as a systematic review. | Title page |
| **ABSTRACT** | | |  |
| Abstract | 2 | See the PRISMA 2020 for Abstracts checklist. | Main manuscript pag 2 |
| **INTRODUCTION** | | |  |
| Rationale | 3 | Describe the rationale for the review in the context of existing knowledge. | Main manuscript pag 3 |
| Objectives | 4 | Provide an explicit statement of the objective(s) or question(s) the review addresses. | Main manuscript pag 3 |
| **METHODS** | | |  |
| Eligibility criteria | 5 | Specify the inclusion and exclusion criteria for the review and how studies were grouped for the syntheses. | Main manuscript pag 4-5 |
| Information sources | 6 | Specify all databases, registers, websites, organisations, reference lists and other sources searched or consulted to identify studies. Specify the date when each source was last searched or consulted. | Main manuscript pag 6 |
| Search strategy | 7 | Present the full search strategies for all databases, registers and websites, including any filters and limits used. | Supplementary Material pag 1 |
| Selection process | 8 | Specify the methods used to decide whether a study met the inclusion criteria of the review, including how many reviewers screened each record and each report retrieved, whether they worked independently, and if applicable, details of automation tools used in the process. | Main manuscript pag 4-5 |
| Data collection process | 9 | Specify the methods used to collect data from reports, including how many reviewers collected data from each report, whether they worked independently, any processes for obtaining or confirming data from study investigators, and if applicable, details of automation tools used in the process. | Main manuscript pag 6-7 |
| Data items | 10a | List and define all outcomes for which data were sought. Specify whether all results that were compatible with each outcome domain in each study were sought (e.g. for all measures, time points, analyses), and if not, the methods used to decide which results to collect. | Main manuscript pag 6-7 |
|  | 10b | List and define all other variables for which data were sought (e.g. participant and intervention characteristics, funding sources). Describe any assumptions made about any missing or unclear information. | Main manuscript pag 6-7 |
| Study risk of bias assessment | 11 | Specify the methods used to assess risk of bias in the included studies, including details of the tool(s) used, how many reviewers assessed each study and whether they worked independently, and if applicable, details of automation tools used in the process. | Main manuscript pag 7 |
| Effect measures | 12 | Specify for each outcome the effect measure(s) (e.g. risk ratio, mean difference) used in the synthesis or presentation of results. | Main manuscript pag 7-8 |
| Synthesis methods | 13a | Describe the processes used to decide which studies were eligible for each synthesis (e.g. tabulating the study intervention characteristics and comparing against the planned groups for each synthesis (item #5)). | Main manuscript pag 7-8 |
|  | 13b | Describe any methods required to prepare the data for presentation or synthesis, such as handling of missing summary statistics, or data conversions. | Main manuscript pag 7-8 |
|  | 13c | Describe any methods used to tabulate or visually display results of individual studies and syntheses. | Main manuscript pag 7-8 |
|  | 13d | Describe any methods used to synthesize results and provide a rationale for the choice(s). If meta-analysis was performed, describe the model(s), method(s) to identify the presence and extent of statistical heterogeneity, and software package(s) used. | Main manuscript pag 7-8 |
|  | 13e | Describe any methods used to explore possible causes of heterogeneity among study results (e.g. subgroup analysis, meta-regression). | Main manuscript pag 7-8 |
|  | 13f | Describe any sensitivity analyses conducted to assess robustness of the synthesized results. | Main manuscript pag 7-8 |
| Reporting bias assessment | 14 | Describe any methods used to assess risk of bias due to missing results in a synthesis (arising from reporting biases). | Main manuscript pag 7-8 |
| Certainty assessment | 15 | Describe any methods used to assess certainty (or confidence) in the body of evidence for an outcome. | Main manuscript pag 7-8 |
| **RESULTS** | | |  |
| Study selection | 16a | Describe the results of the search and selection process, from the number of records identified in the search to the number of studies included in the review, ideally using a flow diagram. | Main manuscript pag 8-9 |
|  | 16b | Cite studies that might appear to meet the inclusion criteria, but which were excluded, and explain why they were excluded. | Main manuscript pag 12 |
| Study characteristics | 17 | Cite each included study and present its characteristics. | Main manuscript pag 9-12 |
| Risk of bias in studies | 18 | Present assessments of risk of bias for each included study. | Main manuscript pag 12-13 |
| Results of individual studies | 19 | For all outcomes, present, for each study: (a) summary statistics for each group (where appropriate) and (b) an effect estimate and its precision (e.g. confidence/credible interval), ideally using structured tables or plots. | Main manuscript (Table 1) pag 39-53 |
| Results of syntheses | 20a | For each synthesis, briefly summarise the characteristics and risk of bias among contributing studies. | Main manuscript pag 13-21 |
|  | 20b | Present results of all statistical syntheses conducted. If meta-analysis was done, present for each the summary estimate and its precision (e.g. confidence/credible interval) and measures of statistical heterogeneity. If comparing groups, describe the direction of the effect. | Main manuscript pag 13-21 |
|  | 20c | Present results of all investigations of possible causes of heterogeneity among study results. | Main manuscript pag 13-21 |
|  | 20d | Present results of all sensitivity analyses conducted to assess the robustness of the synthesized results. | Main manuscript pag 13-21 |
| Reporting biases | 21 | Present assessments of risk of bias due to missing results (arising from reporting biases) for each synthesis assessed. | Main manuscript pag 13-21 |
| Certainty of evidence | 22 | Present assessments of certainty (or confidence) in the body of evidence for each outcome assessed. | Main manuscript pag 13-21 |
| **DISCUSSION** | | |  |
| Discussion | 23a | Provide a general interpretation of the results in the context of other evidence. | Main manuscript pag 22-24 |
|  | 23b | Discuss any limitations of the evidence included in the review. | Main manuscript pag 24 |
|  | 23c | Discuss any limitations of the review processes used. | Main manuscript pag 24 |
|  | 23d | Discuss implications of the results for practice, policy, and future research. | Main manuscript pag 25 |
| **OTHER INFORMATION** | | |  |
| Registration and protocol | 24a | Provide registration information for the review, including register name and registration number, or state that the review was not registered. | Main manuscript pag 4 |
|  | 24b | Indicate where the review protocol can be accessed, or state that a protocol was not prepared. | Main manuscript pag 4 |
|  | 24c | Describe and explain any amendments to information provided at registration or in the protocol. | N/A |
| Support | 25 | Describe sources of financial or non-financial support for the review, and the role of the funders or sponsors in the review. | Funding statement, pag 25 |
| Competing interests | 26 | Declare any competing interests of review authors. | Conflict of interest, pag 26 |
| Availability of data, code and other materials | 27 | Report which of the following are publicly available and where they can be found: template data collection forms; data extracted from included studies; data used for all analyses; analytic code; any other materials used in the review. | Supplementary material |

*From:*  Page MJ, McKenzie JE, Bossuyt PM, Boutron I, Hoffmann TC, Mulrow CD, et al. The PRISMA 2020 statement: an updated guideline for reporting systematic reviews. BMJ 2021;372:n71. doi: 10.1136/bmj.n71

For more information, visit: <http://www.prisma-statement.org/>

**Supplementary Table 7** – List of ongoing studies on Telemonitoring interventions (updated to 5 February 2021).

| **Author, Year, Country** | **Condition** | **Study Title** |
| --- | --- | --- |
| **Omboni 2010**, Italy | Hypertension | Telemonitoring of Blood Pressure in Local Pharmacies (TEMPLAR) |
| **Iversen 2012**, Norway | Diabetes | Telemedicine Follow-up in Primary Health Care for Diabetes-related Foot Ulcers (DiaFOTo) |
| **Colet** **2018,** Spain | Heart Failure | Heart Failure Events Reduction With Remote Monitoring and eHealth Support Investigator Initiated Trial (HERMeS) |
| **Esteban 2018**, Spain | Chronic Obstructive Pulmonary Disease | Impact of the Artificial Intelligence in a Telemonitoring Programme of COPD Patients with Multiple Hospitalizations |
| **Martin 2018**, Germany | Diabetes | TeLIPro Health Program - Active with Diabetes (TeLIPro) |
| **Pekmezaris 2019**, USA | Diabetes | Diabetes Management Program for Hispanic/Latino |
| **Sun 2019**, China | Atrial Fibrillation | Integrative Management of Patients With Atrial Fibrillation Via Hospital-Community-Family-Based Telemedicine (HCFT-AF) Program |
| **Bessonov 2020,** Russia | Acute Coronary Syndrome | Telemedicine Follow-up for Post-ACS Patient |
| **Dominguez** **2020,** Brasil | Cardiovascular disease | Brazilian Heart Insufficiency With Telemedicine (BRAHIT) |
| **Hoffman** **2020,** Germany | Heart Failure | Influence of Telemonitoring on the Management of LVAD-patients |
| **Krzowski** **2020**, Poland | Myocardial Infarction | Mobile App and Digital System for Patients After Myocardial Infarction (afterAMI) |
| **Marinello** **2020**, Italy | Heart Failure | Telemonitoring of Patients Admitted in Hospital at Home With Acute Decompensated Heart Failure - Pilot Study (MONTEROSA) |
| **Naqvi** **2020,** USA | Hypertension | Telehealth After Stroke Care: Integrated Multidisciplinary Access to Post-stroke Care (TASC)s |
| **Nouira** **2020,** Tunisia | Hypertension | The Impact of Telemonitoring in the Management of Hypertension (HOROSCOPE) |
| **Schwartz** **2020,** Uganda | Heart Failure | mHealth for Self-care of Heart Failure in Uganda |
| **Siriwardena** **2020,** UK | Chronic Obstructive Pulmonary Disease | Use of MonitorMe in COPD |
| **Skanes** **2020,** Canada | Atrial Fibrillation | Virtual for Care Atrial Fibrillation Patients Using VIRTUES |
| **Ancker** **2021,** USA | Multiple conditions | Comparative Effectiveness of Telemedicine in Primary Care |
| **Mahler** **2021,** USA | Acute Coronary Syndrome | Enhancing Rural Health Via Cardiovascular Telehealth for Rural Patients Implementation (E-VICTORS) |
| **Noruzbaeva** **2021,** Kyrgyzstan | Coronary Artery Disease | Effects of Remote Monitoring of Patients With Heart Failure Based on Smartphone Application (ERICA-HF) |
| **Prigent 2021,** France | Chronic Obstructive Pulmonary Disease | Study Evaluating Telemonitoring and Experimentation in Telemedicine for the Improvement of Healthcare Pathways (ETAPES Program) Compared to Standard of Care in Patients with Chronic Respiratory Failure Receiving Non-invasive Home Ventilation (e-VENT) |
| **Yin 2021,** China | Diabetes | COVID-19 Lockdown Related Telemedicine for Type 2 Diabetes |

**ACS= Acute Coronary Syndrome; AF=Atrial Fibrillation; CAD= Coronary Artery Disease; HF=Heart Failure; COVID-19;*

**Supplementary Table 8** – Source(s) of Funding for included studies.

| **First author, Year, country** | **Source of Funding** |
| --- | --- |
| **Randomised controlled trials** | |
| **Edmonds 1998**, Canada | N/A |
| **Rogers 2001**, USA | Funded by Welch Allyn, Inc. |
| **Bergenstal 2005**, USA | Grant from Roche Diagnostics Corporation |
| **Cleland 2005**, Germany | Jointly funded by the European Union’s Trans European Network (TEN) and Philips Medical Systems |
| **Shea 2006**, USA | Supported by Cooperative Agreement 95-C-90998 from the Centers for Medicare and Medical Services |
| **Shea 2009**, USA | N/A |
| **Kashem 2008**, USA | Supported by NIH grant no. HL065073; partially supported by a grant from the Commonwealth of Pennsylvania. |
| **Madsen 2008**, Denmark | Grants from Ringkjobing County, the Danish Ministry of Science and the Danish Heart Foundation (07-4-B340-A1446-22372). |
| **Cho 2009**, South Korea | The work was supported by the Ministry of Information and Communication (noninvasive glucose-monitoring project), the Seoul R&D programme and the Ministry of Commerce, Industry and Energy (Energy-IT project). |
| **Dar 2009**, UK | Honeywell HomMed provided the funds to perform this study and the telemonitoring equipment used, via a research contract agreement with Imperial College London. |
| **Giordano 2009**, Italy | Grant of the National Ministry of Health (Contract ICS 030.8/RF00.91). |
| **Istepanian 2009,** UK | Financial and technical support from the IDEN Group, Motorola, USA and the Motohealth team in UK. |
| **Mortara 2009,** UK | supported by E.C. grant (Action line 10.1 ‘Public Health, contract no. QLGA-CT-2001-02424). |
| **Earle 2010,** UK | Financial and technical support from the IDEN Group, Motorola Inc., USA and the Motohealth team in the United Kingdom. |
| **Lewis 2010,** UK | Financial support was provided by an EU grant (C046225). |
| **McManus 2010,** UK | It received joint funding from the Department of Health Policy Research Programme, National Coordinating Centre for Research  Capacity Development, and Midlands Research Practices Consortium (MidReC). Service support costs were obtained from the Department of Health in collaboration with MidReC. Additional funding came from NIHR National School for Primary Care Research. |
| **Bujnowska-Fedak 2011,** Poland |  |
| **Dendale 2012,** Belgium | The Belgian Government Health Insurance Institute (Rijksinstituut voor Ziekte en Invaliditeitsverzekering); Leo Pharma |
| **Konstam 2011,** USA | Supported in part by a research grant from GlaxoSmithKline, Philips Medical Systems, and Health Hero Network. |
| **Neumann 2011,** Germany | It was supported by Sanofi-Aventis Deutschland GmbH |
| **Wade 2011,** USA | Funded by Aetna, Inc, and Intel, Inc. |
| **Blasco 2012,** Spain | It was supported by the Ministry of Health and Consumer Affairs of Spain (grant number PI[051882]), by the National Scientific Research, Development and Technological Innovation Plan (grant number TSI2005-02682), and by the MOBIS Program of the  Spanish Vodafone Foundation. |
| **Boyne 2012,** Netherlands | Funding from The Province of Limburg in The Netherlands; the Annadal Foundation Maastricht, Astra Zeneca [an unrestricted grant]; the Rescar Foundation Maastricht, The Netherlands. |
| **Dinesen 2012,** Denmark | N/A |
| **Seto 2012,** Canada | Partly funded by the Toronto General Hospital Foundation and the Natural Sciences and Engineering Research Council of Canada Strategic Research Network Grant entitled Healthcare Support through Information Technology Enhancements (hSITE). |
| **De San Miguel 2013,** Australia | Funded by the Australian Department of Health and Ageing |
| **Kerry 2013,** UK | Funded by The Stroke Association grant no. TSA 2006/05 (main study) and by the Isaak Schapera Research Trust (feasibility study). |
| **Madigan 2013,** USA | Fundend by the Case Western Reserve University/Cleveland Clinic CTSA Grant Number UL1 RR024989 from the National Center for Research Resources (NCRR). |
| **Margolis 2013, 2018,** USA | Funded by grant 2-R01-HL090965 from the National Heart, Lung and Blood Institute |
| **McKinstry 2013,** UK | Funded by the BUPA Foundation (grant No 748/G24) with additional support from the High Blood pressure Foundation and NHS Lothian |
| **Bentley 2014,** UK | N/A |
| **Blum 2014,** USA | Financial support from Medicare Coordinated Care Demonstration Project. |
| **Pressman 2014,** USA | This project was partially funded by a grant from the Samsung  Group, Inc. |
| **Ralston 2014,** USA | N/A |
| **Villani 2014,** Italy | Grant Italian Ministry of Research and Public Instruction (FIRB RBNE01KYE4 2003). |
| **Vourinen 2014,** Finland | Funded by The Finnish Funding Agency for Technology and Innovation and VTT Technical Research Centre of Finland |
| **Fountoulakis 2015,** Greece | N/A |
| **Greenwood 2015,**  USA | Financial support from from the Investigator Initiated Studies program of LifeScan Corporation, Intel-GE Care Innovations, Sutter Institute for Medical Research, The Betty Irene Moore School of Nursing, The Jonas Center for Nursing Excellence, and the University of California Davis, National Center for Advancing Translational Sciences, National Institutes of Health, through grant number UL1 TR 000002. |
| **Varon 2015,** UK | Research supported by EU: RECAP 209G within INTERREG IVB NWE programme |
| **Evans 2016,** USA | Funded by a grant from the National Institute on Aging, ‘‘Non-intrusive Automated Portable- Collection Systems for Aging Surveys II,’’ SBIR Phase II grant 2R44AG02196-02. |
| **Kardas 2016,** Poland | Study partially supported by the EU FP7 Project COMMODITY12 (www.commodity12.eu; grant agreement no:287841). |
| **Ong 2016,** USA | This study was supported by grant R01HS019311 fromthe Agency for Healthcare Research and Quality; by grant RC2HL101811 from  the National Heart, Lung, and Blood Institute (NHLBI); by grant UL1TR000124 fromthe National Center for Advancing Translational Science (NCATS) of the University of California, Los Angeles, Clinical and Translational Science Institute; by grant 66336 fromthe RobertWood Johnson Foundation; by the Sierra Health Foundation; by the University of California Center for Health Quality and Innovation; and by the participating institutions. |
| **Vianello 2016,** Italy | Funded by the EU Commission (Grant Agreement No 250187) |
| **Wild 2016,** UK | Funded by a Chief Scientist Office Applied Research Programme Grant (ARPG/07/3) (http://www.cso.scot.nhs.uk/) |
| **Baron 2017, 2017b,** UK | Funded by the Policy Research Programme of the Department of Health for England. |
| **Beran 2018,** USA | Funded by a grant from the National Heart, Lung, and Blood Institute (R01HL090965) |
| **Dang 2017,** USA | Funded by the Florida Department of Health’s James and Esther King Biomedical Research Program, grant number 09KC-01. |
| **Dario 2017,** Italy | Study co-founded by the European Commission and 21 partners from nine European regions in the context of the REgioNs of EuropeWorkINg toGether for HEALTH (RENEWING HEALTH) project. |
| **Egede 2017,** USA | Funded by Grant No. W81XWH-10-2-0057 from the Department of Defense |
| **Gallagher 2017,** USA | This work was supported by the New York-Presbyterian Hospital 2014 Translational Grants Program. |
| **Frederix 2018,** Belgium | This work was supported by the Research Foundation Flanders  (FWO) [grant number 1128915N]. |
| **Koehler 2018,** Germany | This work was supported by a research grant of the German Federal Ministry of Education and Research (grant numbers 13KQ0904A, 13KQ0904B, 13KQ1104A). |
| **Kotooka 2018,** Japan | Funded by grants from the Japanese Ministry of Health, Labor, and Welfare Comprehensive Research on Aging and Health (KR23000003, KR24000001), and the Japan Society for the Promotion of Science KAKENHI Grant Number JP17K09510. |
| **Soriano 2018,** Spain | Funded by the Fundació n Teófilo Herando, Universidad Autónoma de Madrid, with the support of Linde Healthcare. |
| **Tupper 2018,** Denmark | N/A |
| **Valdivieso 2018,** Spain | N/A |
| **Walker 2018,** Spain | Funded by a European Commission grant (no. 306093) within the call identifier FP7-HEALTH-2012-INNOVATION-1. |
| **Nouryan 2019,** USA | Funded by grants from the Fan Fox & Leslie R. Samuels Foundation and the Verizon Foundation. |
| **Cichosz 2020,** Denmark | Funded by North Denmark Region, the 11 municipalities in North Denmark Region, and the Danish Agency for Digitalization Policy and Strategy. |
| **Non-randomised studies** | |
| **de Lusignan 2001,** UK | Funded by MSD Pharmaceuticals and Agilent |
| **Tsang 2001,** Hong Kong | Funded by a grant of the Health Services Research Committee (HRSC#722006). |
| **Trudel 2007,** Canada | Funded by the Primary Care Health Transition Fund of the Ministry of Health and Long Term Care of Ontario. |
| **Antonicelli 2008,** Italy | Funded by the Italian Ministry of Health. |
| **Antonicelli 2010,** Italy | N/A |
| **Kim 2008,** South Korea | Korea Research Grant funded by the Korean Government (MOEHRD) (KRF-2005-015- E00232). |
| **Rodriguez-Idigoras 2009,** Spain | Funded by the Roche Diagnostics Spain (Diabetes Care) |
| **Sicotte 2011,** Canada | Funded by the Canadian Institutes of Health Research (CIHR) |
| **Stuckey 2011,** Canada | Funded by the Canadian Institutes of Health Research, the Canadian Diabetes Association, the Heart and Strok Foundation for the Team Canada and Finland (2007-2012) grant 83029. |
| **Chau 2012,** Hong Kong | N/A |
| **Domingo 2012,** Spain | Funded by Philips Healthcare and by the Catalan Institute of Health. |
| **Karg 2012,** Germany | N/A |
| **Agboola 2013,** USA | Supported by the Verizon Foundation. |
| **Chen 2013,** Taiwan | Funded by the Taiwan National Science Council (grant numbers  NSC 100-2220-E-002-020 and NSC 99-2911-I-008-100), by Taiwan University (grant number 10R71608-1), and the Department of Health, Executive Yuan, ROC (grant number DOH 99-TD-B-111-001) |
| **Bernocchi 2014,** Italy | N/A |
| **Mira-Solves 2014,** Spain | Telefónica de España S.A. |
| **Schoenfeld 2014,** USA | Funded by Medtronic, Inc. Minneapolis, Minnesota, USA |
| **DeAlleaume 2015,** USA | Funded by the Colorado Department of Public and Environment Cancer, Cardiovascular, and Pulmonary Disease Program (contract no. 09FLA00292) |
| **Dierckx 2015,** UK | Financial support by Philips |
| **Evangelista 2015,** USA | Funded by the National Heart, Lung, and Blood Institute (1R01HL093466-01) and University of California, Los Angeles, Resource Centers for Minority Aging Research/Center for Health Improvement of Minority Elderly, under National Institute in Aging (P30-AG02-1684) |
| **Hanley 2015,** UK | Funded by the Scottish Government Chief Scientist Office |
| **Donate-Martinez 2016,** Spain | Funded by the Agencia Valenciana de Salud of Ministry of Health of Valencia (2011) and from the Valencian Government through the project Prometeo-OpDepTec Fase II (Project reference: PROMETEUII/2014/074) |
| **Grady 2016,** UK | Funded by LifeScan, Inc. |
| **Amir 2017,** Israel | Sponsored by SensibleMedical Innovations Ltd. |
| **Nissen 2017,** Denmark | N/A |
| **Orozco-Beltran 2017,** Spain | Devices provided by Telefonica España SA |
| **Lee 2018,** UK | Funded by the National Institute for Health Research (NIHR) Collaboration for Leadership in Applied Health Research and Care Northwest London (NIHR CLAHRC NWL). |
| **Lee 2019,** Malaysia | Funded by the e-Science fund from the Ministry of Science, Technology and Innovation, Malaysia (03-02-10-SF0238 (MOSTI)),  Telemedicine Cluster, Tropical Medicine and Biology platform, Monash University Malaysia (52140757-314-00) and SEGi University Research Fund (SEGi/2013/SKK/04/1). |
| **Michaud 2018,** USA | Funded by the Department of Health and Human Services, Centers for Medicare & Medicaid Services (grant number 1C1CMS331344) |
| **Grant 2019,** UK | Funded by an National Institute for Health Research  (NIHR) Programme Grant for Applied Heath Research (grant reference: RP-PG-1209-10051) and by an NIHR professorship  awarded to Richard J McManus (reference number: NIHR-RP-R2-12-015). |
| **van Berkel 2019,** UK | None |
| **Buis 2020,** USA | Funded by the Michigan Institute for Clinical & Health Research (UL1TR000433). |
| **Leng Chow 2020,** Singapore | Funded by the EDB, Singapore Living Lab Fund and Philips Electronics – Hospital to Home Pilot Project (EDB grant reference number: S14-1035-RF-LLF H and W). |
| **Pekmezaris 2020,** USA | Funded by the Patient-Centered Outcomes Research Institute (PCORI), award #AD-2017C3-9185 |
