# Supplementary material for: Interactive Remote Patient Monitoring Devices for Managing Chronic Health Conditions: Systematic Review and Meta-analysis
Source: J Med Internet Res. 2022 Nov 3;24(11):e35508. doi: 10.2196/35508 (PMC9673001; doi:10.2196/35508)
Supplement: Multimedia Appendix 2 [file jmir_v24i11e35508_app2.docx]

**Multimedia Appendix 2.** Summary of the included studies (N=96).

| Study type and authors, year, and country | | Study population, N | Condition | Intervention type, number of participants, age (years), and men (n [%]) | Comparator, number of participants, age (years), and men (n [%]) | Outcomes | Follow-up | Impact of telemonitoring |
| --- | --- | --- | --- | --- | --- | --- | --- | --- |
| **Randomized controlled trials** | | | | | | | | |
|  | Edmonds et al [44], 1998, Canada | 35 | Type 2 diabetes | Mobile phone data transmission, 16, not reported, not reported | Usual care, 19, not reported, not reported | Adherence and satisfaction | 3 months | Further studies required |
|  | Rogers et al [90], 2001, United States | 121 | Hypertension | Manual uploading of BP^a^ values on a web-based platform, 60, mean 62.6 (SD 10.0), 43 men; 57 women^b^ | Usual care, 6, Mean 60.3 (SD 11.9), 58 men; 42 women^b^ | Changes in BP | 2 to 3 months | +^c^ |
|  | Bergenstal et al [22], 2005, United States | 47 | Type 2 diabetes | Automated data transmitted via modem, 24, mean 44 (SD 17), 37 men; 63 women^b^ | Data transmitted via telephone, 23, mean 45 (SD 13), 39 men; 61 women^b^ | HbA1c^d^ and satisfaction | 4 weeks | + |
|  | Cleland et al [32], 2005, Germany | 299 | CHF^e^ | Home telemonitoring; automated data collection and transmission via dedicated device, 163, mean 67 (SD 13), 45 men; 55 women^b^ | Nurse telephone support and usual care; data transmission via telephone and usual care, nurse telephone support: 170; usual care: 85, nurse telephone support: mean 68 (SD 10); usual care: mean 67 (SD 11), nurse telephone support: 73; usual care: 82 | ACM^f^ | 240 days | −^g^ |
|  | Shea et al [93,94], 2006 and 2009, United States | 1665 | Type 2 diabetes | Manual upload of data on dedicated device or software, 844, mean 70 (SD not specified), not reported | Usual care, 821, mean 70 (SD not specified), not reported | BP and HbA1c percentage | 12 months | + |
|  | Kashem et al [61], 2006, United States | 36 | HF^h^ | Manual upload of data on dedicated device or software, 18, 55.1 (SD 12.6), men: 12 (67), women: 6 (33) | Usual care, 18, mean 52.2 (SD 10.6), men: 13 (72); women: 5 (28) | Hospitalization | 6 months | + |
|  | Kashem et al [60], 2008, United States | 48 | HF^h^ | Manual upload of data on dedicated device or software, 24, mean 53 (SD 10), 72 men; 28 women^b^ | Usual care, 24, mean 54 (SD 10), 76 men; 24 women^b^ | Mortality and hospitalization | 12 months | =^i^ |
|  | Madsen et al [72], 2008, Denmark | 136 | Hypertension | Automated upload of data on dedicated device or software, 113, mean 55 (SD 11.7), men: 55 (49); women: 58 (61) | Usual care, 123, mean 56.7 (SD 11.6), men: 64 (53); women: 59 (36) | BP | 6 months | = |
|  | Cho et al [30], 2009, South Korea | 69 | Type 2 diabetes | Mobile app, 35, mean 51.1 (SD 13.1), 26 men; 74 women^b^ | Web-based telemonitoring system, 34, mean 51.1 (SD 13.1), 26 men; 74 women^b^ | HbA1c percentage, satisfaction, and adherence | 3 months | = |
|  | Dar et al [34], 2009, United Kingdom | 299 | CHF | Automated upload of data on dedicated device or software, 84, mean 72 (SD 12), men: 62 (74); women: 29 (26) | Usual care, 89, mean 72 (SD 12), men: 59 (66); women: 32 (44) | Number of nonelective hospitalizations and number of HF-related admissions | 6 months | = |
|  | Giordano et al [52], 2009, Italy | 460 | CHF | Automated upload of data on dedicated device or software, 230, mean 57 (SD 10), men: 214 (93); women: 16 (7) | Usual care, 230, mean 57 (SD 10), men: 216 (94); women: 14 (6) | Number of HF-related hospitalizations | 12 months | + |
|  | Istepanian et al [57], 2009, United Kingdom | 137 | Type 2 diabetes | Automated upload of data on dedicated device or software, 72, mean 60 (SD 12), not specified | Usual care, 65, mean 57 (SD 13), not specified | HbA1c | 9 months | Not clear |
|  | Mortara et al [80], 2009, United Kingdom | 461 | CHF | Divided as follows: monthly telephone contact; monthly telephone contact+data transmission; monthly telephone contact+data transmission+24-hour cardiorespiratory recording; answering machine+nurse telephone support+weekly data transmission, 301 (monthly telephone contact: 106; monthly telephone contact+data transmission: 94; monthly telephone contact+data transmission+24-hour cardiorespiratory recording: 101), mean 60 (SD 12), not specified | Usual care, 160, mean 60 (SD 12), not specified | ACH^j^, combined end point, number of cardiac deaths, and number of HF-related hospitalizations | 12 months | + |
|  | Earle et al [45], 2010, United Kingdom | 137 | Type 2 diabetes and hypertension | Automated upload of data on dedicated device or software, 72, mean 59.6 (SD 12), not specified | Usual care, 65, mean 57.1 (SD 13), not specified | BP | 6 months | + |
|  | Lewis et al [70], 2010, United Kingdom | 40 | COPD^k^ | Manual upload of data on dedicated device or software, 20, Median 70 (IQR 61-73), 50 men; 50 women^b^ | Usual care, 20, median 73 (IQR 63-79), 50 men; 50 women^b^ | QoL^l^ | 6 months | = |
|  | McManus et al [77], 2010, United Kingdom | 527 | Hypertension | Automated upload of data on dedicated device or software, 263, mean 66.2 (SD 8.8), men: 110 (47); women: 152 (53) | Usual care, 264, mean 66.2 (SD 8.8), men: 115 (47); women: 149 (53) | BP | 6 months | + |
|  | Bujnowska-Fedak et al [27], 2011, Poland | 100 | Type 2 diabetes | Manual upload of data on dedicated device or software, 50, mean 53.1 (SD 25.2), 52 men; 48 women^b^ | Usual care, 50, mean 57.5 (SD 27.4), 50 men; 50 women^b^ | HbA1c | 3 months | + |
|  | Dendale et al [39], 2011, Belgium | 160 | CHF | Manual upload of data on dedicated device or software, 80, mean 76 (SD 10), men: 50 (62); women: 30 (48) | Usual care, 80, mean 76 (SD 10), men: 54 (67); women: 13 (33) | ACM, number of HF-related hospitalizations, and number of hospitalizations | 6 months | + |
|  | Konstam et al [65], 2011, United States | 88 | HF | Manual upload of data on dedicated device or software, 44, mean 71.7 (SD 12), men: 26 (59); women: 18 (41) | Usual care, 44, mean 67 (SD 13.1), men: 30 (68); women: 14 (32) | QoL (MLHFQ^m^) | 6 weeks | = |
|  | Neumann et al [81], 2011, Germany | 60 | Hypertension | Automated upload of data on dedicated device or software, 30, mean 54.7 (SD 17.4), men: 13 (43); women: 17 (57) | Usual care, 30, mean 56.2 (SD 17.4), men: 16 (53); women: 14 (47) | BP (24 hours) | 3 months | + |
|  | Wade et al [107], 2011, United States | 316 | CHF | Automated upload of data on dedicated device or software, 164, mean 78.1 (SD not reported), 51 men; 49 women^b^ | Usual care, 152, mean 78.1 (SD not reported), 53 men; 47 women^b^ | Mortality, QoL, and number of cardiovascular-related hospitalizations | 6 months | = |
|  | Blasco et al [24], 2012, Spain | 203 | Acute coronary syndrome | Manual transmission of data via mobile phone, 102, mean 60.6 (SD 11.5), men: 83 (81); women: 19 (19) | Usual care, 101, mean 61 (SD 12.1), men: 80 (79); women: 21 (21) | BP | 12 months | + |
|  | Boyne et al [25], 2012, Netherlands | 328 | CHF | Telephone support+usual care, 197, mean 71 (SD 11), men: 115 (58); women: 82 (42) | Usual care, 185, mean 71 (SD 11), men: 111(60); women: 74 (40) | Number of HF-related hospitalizations and number of cardiovascular-related hospitalizations | 12 months | Further studies required |
|  | Dinesen et al [41], 2012, Denmark | 111 | COPD | Manual upload of data on dedicated device or software, 61, median 68 (IQR 45-82), not reported | Usual care, 51, median 68 (IQR 45-82), not reported | Number of hospital admissions | 10 months | + |
|  | Seto et al [92], 2012, Canada | 100 | CHF | Automated upload of data on dedicated device or software, 50, mean 55.1 (SD 13.7), men: 41 (82); women: 9 (18) | Usual care, 50, mean 52.3 (SD 13.7), men: 38 (76); women: 12 (24) | QoL (MLHFQ), number of hospitalizations, mortality rate, and adherence | 6 months | + |
|  | De San Miguel et al [37], 2013, Australia | 71 | COPD | Automated upload of data on dedicated device or software, 35, mean 74 (SD not reported), men: 20 (57); women: 15 (43) | Usual care, 36, mean 71 (SD not reported), men: 14 (39); women: 22 (61) | Number of hospitalizations and BP | 6 months | = |
|  | Kerry et al [62], 2013, United Kingdom | 318 | Hypertension | Telephone support+manual data transmission, 187, mean 71.1 (SD 12.6), men: 111 (59.4); women: 76 (40.6) | Usual care, 194, mean 71.1 (SD 12.6), men: 108 (55); women: 86 (45) | BP and QoL | 6 months | = |
|  | Madigan et al [71], 2013, United States | 514 | HF | Automated upload of data on dedicated device or software, 54, mean 75 (SD 12.1), men: 14 (26); women: 40 (74) | Usual care, 45, mean 74.7 (SD 11.3), men: 18 (40); women: 27 (60) | Number of rehospitalizations | 180 days | = |
|  | Margolis et al [73,74], 2013 and 2018, United States | 450 | Hypertension | Automated upload of data on dedicated device or software, 228, mean 61.1 (SD 12), men: 125 (54.8); women: 103 (45.2) | Usual care, 222, mean 61.1 (SD 12), men: 121 (55.0); women: 101 (45) | BP | 6 months | + |
|  | McKinstry et al [75], 2013, United Kingdom | 401 | Hypertension | Manual upload of data on dedicated device or software, 200, mean 60.5 (SD 11.8), men: 117 (58.5); women: 83 (41.5) | Usual care, 201, mean 60.8 (SD 10.7), men: 120 (60); women: 81 (40) | BP | 6 months | + |
|  | Bentley et al [20], 2014, United Kingdom | 63 | COPD | Automated upload of data on dedicated, device or software, 32, mean 66.6 (SD 10.5), men: 12 (36); women: 20 (44) | Usual care, 31, mean 66.6 (SD 10.5), men: 11 (36); women: 20 (44) | Number of hospital admissions, QoL, and mortality | 2 months | Further studies required |
|  | Blum and Gottlieb [23], 2014, United States | 203 | CHF | Automated upload of data on dedicated device or software, 102, mean 73 (SD 8), not specified | Usual care, 101, mean 72 (SD 10), not specified | Number of hospitalizations and QoL (SF-36^n^) | 6 months | = |
|  | Pressman et al [87], 2014, United States | 225 | Type 2 diabetes | Manual upload of data on dedicated device or software, 118, mean 55.2 (SD 9.3), men: 67 (63); women: 51 (47) | Usual care, 107, mean 56.4 (SD 8.7), men: 55 (60); women: 552 (40) | HbA1c and BP | 6 weeks | = |
|  | Ralston et al [88], 2014, United States | 778 | Hypertension | BP monitor+pharmacist support, 261, mean 59.8 (SD 8.9), 56 men; 44 women^b^ | BP monitor only and usual care, BP monitor only: 259; usual care: 258, BP monitor only: mean 59.8 (SD 8.3); usual care: not reported, BP monitor only: 45; usual care: not reported | BP | 12 months | + |
|  | Villani et al [105], 2014, Italy | 80 | CHF | Manual upload of data on dedicated device or software, 40, mean 72 (SD 3), men: 30 (75); women: 10 (25) | Usual care, 40, mean 72 (SD 3), men: 29 (72); women: 11 (28) | Mortality rate, number of hospitalizations for HF, and QoL (PHQ^o^) | 12 months | + |
|  | Vuorinen et al [106], 2014, Finland | 94 | HF | Manual upload of data on dedicated device or software, 47, mean 58.3 (SD 11.6), men: 39 (83); women: 8 (17) | Usual care, 47, mean 57.9 (SD 11.9), men: 39 (83); women: 8 (17) | Number of hospital admissions | 6 months | = |
|  | Fountoulakis et al [49], 2015, Greece | 105 | Type 2 diabetes | Automated upload of data on dedicated device or software, 70, mean 55.2 (SD 16.1), men: 45 (64); women: 25 (36) | Usual care, 35, mean 55.4 (SD 16.1), men: 24 (68); women: 11 (32) | HbA1c | 3 months | + |
|  | Greenwood et al [55], 2015, United States | 90 | Type 2 diabetes | Manual upload of data on dedicated device or software, 45, mean 58 (SD 11), 75 men; 25 women^b^ | Usual care, 45, mean 58 (SD 11), 79 men; 21 women^b^ | HbA1c | 3 months | + |
|  | Varon et al [103], 2015, United Kingdom | 534 | AF^p^ | Automated upload of data on dedicated device or software (device 1), 399, mean 63.1 (SD 12.6), not specified | Automated upload of data on dedicated device or software (device 2), 135, mean 63.1 (SD 12.6), not specified | Compliance and QoL (EQ-5D-3L) | 6 weeks | + |
|  | Evans et al [48], 2016, United States | 441 | HF and healthy | Disease group: automated upload of data on dedicated device or software, 21, mean 71.8 (SD 8.8), 46 men; 54 women^b^ | Healthy group: automated upload of data on dedicated device or software, 20, mean 72.2 (SD 4.3), 50 men; 50 women^b^ | Adherence | 6 months | + |
|  | Kardas et al [58], 2016, Poland | 60 | Type 2 diabetes | Automated upload of data on dedicated device or software, 30, mean 59.9 (SD 5.31), men: 17 (57); women: 13 (43) | Usual care, 30, mean 59 (SD 8.9), men: 19 (63); women: 11 (37) | QoL, HbA1c, BP, and adherence | 6 weeks | + |
|  | Ong et al [84], 2016, United States | 1437 | CHF | Automated upload of data on dedicated device or software, 715, mean 73 (SD not reported), men: 382 (53.4); women: 333 (46.6) | Usual care, 722, mean 73 (SD not reported), men: 382 (53.4); women: 333 (46.6) | ACM, ACH, QoL (MLHFQ), and adherence | 1 month | = |
|  | Vianello et al [104], 2016, Italy | 334 | COPD | Manual upload of data on dedicated device or software, 230, mean 75.96 (SD 6.54), men: 164 (71.3); women: 66 (28.7)^b^ | Usual care, 104, mean 48 (SD 6.16), men: 76 (73.1); women: 28 (26.9)^b^ | QoL and number of hospital admissions | 12 months | = |
|  | Wild et al [109], 2016, United Kingdom | 321 | Type 2 diabetes | Automated upload of data on dedicated device or software, 160, mean 61 (SD 9.8), men: 106 (66.3); women: 54 (33.7) | Usual care, 161, mean 61 (SD 9.8), men: 108 (67.1); women: 53 (32.9) | HbA1c and BP | 9 months | + |
|  | Baron et al [18,19], 2017, United Kingdom | 81 | Type 2 diabetes | Automated upload of data on dedicated device or software, 45, mean 58.2 (SD 13.6), men: 31 (69); women: 14 (31) | Usual care, 36, mean 55.6 (SD 13.8), men: 15 (43); women: 21 (57) | HbA1c and BP | 9 months | = |
|  | Beran et al [21], 2018, United States | 450 | Hypertension | Automated upload of data on dedicated device or software, 228, mean 61.1 (SD 12), 55 men; 45 women^b^ | Usual care, 222, mean 61.1 (SD 12), 55 men; 45 women^b^ | BP | 6 months | + |
|  | Dang et al [136], 2017, United States | 61 | CHF | Questionnaires via mobile phone, 42, mean 55 (SD 9.8), men: 27 (64); women: 15 (36) | Usual care, 19, mean 55 (SD 9.8), men: 11 (63); women: 7 (37) | QoL (MLHFQ and GHQ^q^) | 3 months | + |
|  | Dario et al [35], 2017, Italy | 299 | Type 2 diabetes | Manual upload of data on dedicated device or software, 208, mean 73 (SD 5.8), men: 119 (57); women: 87 (43) | Usual care, 91, mean 73 (SD 5.3), men: 49 (53); women: 42 (47) | QoL (SF-36) and HbA1c | 12 months | Further studies required |
|  | Egede et al [46], 2017, United States | 113 | Type 2 diabetes | Manual upload of data on dedicated device or software, 54, mean 54.2 (SD 11), 19 men; 81 women^b^ | Usual care, 59, mean 54.2 (SD 11), 19 men; 81 women^b^ | HbA1c | 3 months | + |
|  | Gallagher et al [51], 2017, United States | 40 | HF | Automated upload of data on dedicated device or software, 20, median 86 (IQR 50-77), men: 15 (75); women: 5 (25) | Usual care, 20, median 86 (IQR 50-77), men: 15 (75); women: 5 (25) | Adherence and number of hospital readmissions | 1 month | + |
|  | Frederix et al [50], 2018, Belgium | 142 | CHF | Manual upload of data on dedicated device or software, 77, mean 76 (SD 10), men: 49 (64); women: 28 (36) | Usual care, 66, mean 76 (SD 10), men: 44 (67); women: 22 (33) | ACM | 6 months | = |
|  | Koehler et al [64], 2018, Germany | 1571 | CHF | Manual upload of data on dedicated device or software, 765, mean 70 (SD 10), men: 533 (69.7); women: 232 (40.3) | Usual care, 773, mean 70 (SD 10), men: 537 (69.5); women: 236 (30.5) | ACM and cardiovascular mortality | 12 months | + |
|  | Kotooka et al [66], 2018, Japan | 183 | CHF | Automated upload of data on dedicated device or software, 93, mean 67.1 (SD 12.8), men: 51 (56); women: 39 (44) | Usual care, 91, mean 65.4 (SD 15.6), men: 56 (61); women: 35 (39) | ACM, cardiovascular mortality, ACH, cardiovascular rehospitalizations, and adherence | 1 month | = |
|  | Soriano et al [96], 2018, Spain | 229 | COPD | Automated upload of data on dedicated device or software, 115, mean 71 (SD 8), men: 92 (80); women: 23 (20) | Usual care, 114, mean 71 (SD 8), men: 91 (80); women: 23 (20) | ACM, ACH, and QoL (EQ-5D) | 12 months | = |
|  | Tupper et al [100], 2018, Denmark | 281 | COPD | Manual upload of data on dedicated device or software, 141, mean 69.8 (SD 9), men: 55 (39); women: 86 (61) | Usual care, 140, mean 69.4 (SD 10), men: 76 (55); women: 64 (45) | QoL (15D^r^) | 6 months | + |
|  | Valdivieso et al [101], 2018, Spain | 427 | Chronic conditions (COPD, type 2 diabetes, and HF) | Manual upload of data on dedicated device or software, 95, mean 69.8 (SD not reported), 71 men; 29 women^b^ | Telephone support and usual care, telephone support: 179; usual care: 198, telephone support and usual care: mean 75. 9 (SD not reported), telephone support: 51; usual care: 54 | QoL (EQ-5D), mortality, and number of hospital admissions | 12 months | + |
|  | Walker et al [108], 2018, Spain | 312 | COPD | Manual upload of data on dedicated device or software, 154, median 71 (IQR not reported), 44 men; 66 women^b^ | Usual care, 158, median 71 (IQR not reported), 44 men; 66 women^b^ | QoL (EQ-5D) | 9 months | = |
|  | Nouryan et al [83], 2019, United States | 98 | HF | Automated upload of data on dedicated device or software, 42, mean 81.4 (SD not reported), 32 men; 68 women^b^ | Usual care, 47, mean 84.9 (SD not reported), 32 men; 68 women^b^ | Number of hospitalizations and QoL (MLHFQ) | 6 months | + |
|  | Cichosz et al [31], 2020, Denmark | 299 | CHF | Disease-specific questionnaires via tablet, 145, median 70 (IQR 59.5-77), 57 men; 43 women^b^ | Usual care, 154, median 69 (IQR 61-76), 51 men; 49 women^b^ | QoL (SF-36) | 12 months | = |
| **Nonrandomized studies** | | | | | | | | |
|  | De Lusignan et al [36], 2001, United Kingdom | 20 | CHF | Manual upload of data on dedicated device or software, 10, mean 75.2 (SD not reported), not reported | Usual care, 10, mean 75.2 (SD not reported), not reported | Adherence and QoL (GHQ) | 12 months | + |
|  | Tsang et al [99], 2001, Hong Kong | 19 | Type 2 diabetes | Electronic diary+health questionnaires, 10, mean 30 (SD 8), 50 men; 50 women^b^ | Usual care, 9, mean 35 (SD 8), 70 men; 30 women^b^ | HbA1c | 3 months | + |
|  | Schoenfeld et al [91], 2004, United States | 59 | CHF | Manual upload of data on dedicated device or software, 59, mean 64 (SD 14), men: 45 (76); women: 14 (24) | N/A^s^ | Satisfaction | 7 days | + |
|  | Trudel et al [98], 2007, Canada | 30 | Type 2 diabetes and hypertension | Mobile app acting as personal medical diary, 30, not specified, not specified | N/A | BP | 4 months | Further investigation required |
|  | Antonicelli et al [15,16], 2008 and 2010, Italy | 57 | CHF | Manual upload of data on dedicated device or software, 28, mean 78 (SD 7), men: 16 (57); women: 12 (43) | Usual care, 29, mean 78 (SD 7), men: 19 (65); women: 10 (35) | Combined rate of H&M^u^, QoL, and BP | 12 months | + |
|  | Kim and Kim [63], 2008, South Korea | 34 | Type 2 diabetes | Automated upload of data on dedicated device or software+SMS text messaging, 18, mean 45.5 (SD 9.1), men: 9 (50); women: 9 (50) | Usual care, 16, mean 48.5 (SD 8.0), men: 7 (44); women: 9 (56) | HbA1c percentage | 3 months | + |
|  | Rodriguez-Idigoras and Sepulveda-Munoz [89], 2009, Spain | 328 | Type 2 diabetes | Manual upload of data on dedicated device or software, 116, mean 63 (SD not reported), men: 87 (54); women: 29 (46) | Usual care, 167, mean 64 (SD not reported), men: 82 (49); women: 85 (51) | HbA1c percentage | 12 months | + |
|  | Sicotte et al [95], 2011, Canada | 46 | COPD | Manual upload of data on dedicated device or software, 23, mean 73.7 (SD 9.6), men: 13 (56); women: 10 (44) | Usual care, 23, mean 75.4 (SD 9.7), men: 13 (56); women: 10 (44) | Satisfaction and QoL (SF-12^u^) | 3 months | = |
|  | Stuckey et al [97], 2011, Canada | 24 | Cardiovascular disease or type 2 diabetes | Manual upload of data on dedicated device or software, 24, mean 56.6 (SD 8.9), men: 6 (25); women: 18 (75) | N/A | BP and compliance | 8 weeks | + |
|  | Chau et al [28], 2012, Hong Kong | 40 | COPD | Manual upload of data on dedicated device or software, 22, mean 73.5 (SD 6), men: 21 (95); women: 1 (5) | Usual care, 18, mean 72.2 (SD 6), men: 18 (100); women: 0 (0) | Satisfaction, QoL (CRQ^v^), and number of hospitalizations | 2 months | + |
|  | Domingo et al [42], 2012, Spain | 97 | HF | Automated upload of data on dedicated device or software, 46, mean 66.5 (SD 11.5), men: 14 (30); women: 32 (70) | Usual care, 51, mean 66.5 (SD 11.5), men: 15 (30); women: 36 (70) | Adherence and satisfaction | 6 months | + |
|  | Karg et al [59], 2012, Germany | 36 | COPD | Automated upload of data on dedicated device or software, 36, mean 67.9 (SD 6.9), men: 27 (75); women: 9 (25) | N/A | Adherence | 6 months | + |
|  | Agboola et al [13], 2013, United States | 30 | Hypertension | Web-based device, 15, mean 61.9 (SD not reported), 20 men; 80 women^b^ | Mobile BP device, 15, mean 61.6 (SD not reported), 20 men; 80 women^b^ | Adherence | 2 months | + |
|  | Chen et al [29], 2013, Taiwan | 141 | Cardiovascular disease | Automated upload of data on dedicated device or software, 141, median 70.8 (IQR 60.8-78.3), men: 86 (61); women: 55 (39) | N/A | ACH | 6 months | + |
|  | Bernocchi et al [17], 2014, Italy | 168 | Hypertension | Automated upload of data on dedicated device or software, 74, mean 59.7 (SD 12.5), men: 38 (51); women: 36 (49) | Usual care, 94, mean 59.1 (SD 13.3), men: 50 (53); women: 44 (47) | BP | 2 to 4 months | + |
|  | Mira-Solves et al [78], 2014, Spain | 410 | Chronic conditions (type 2 diabetes, hypertension, CHF, and COPD) | Automated upload of data on dedicated device or software, 410, not reported, 64 men; 36 women | N/A | Satisfaction | 24 months | + |
|  | DeAlleaume et al [38], 2015, United States | 1289 | Hypertension | Automated upload of data on dedicated device or software, 1289, mean 60.3 (SD not reported), 59 men; 41 women^b^ | N/A | BP | 12 months | + |
|  | Dierckx et al [40], 2015, United Kingdom | 278 | HF | Automated upload of data on dedicated device or software, 278, mean 71 (SD 12), men: 243 (73); women: 35 (27) | N/A | Mortality and rehospitalization rate | 6 months | + |
|  | Evangelista et al [47], 2015, United States | 42 | HF | Manual upload of data on dedicated device or software, 21, mean 72.7 (SD 8.9), men: 10 (48); women: 11 (52) | Usual care, 21, mean 72.7 (SD 8.9), men: 10 (48); women: 11 (52) | QoL (MLHFQ) | 3 months | + |
|  | Hanley et al [56], 2015, United Kingdom | 23 | Type 2 diabetes | Automated upload of data on dedicated device or software, 23, mean 60 (SD not reported), men: 16 (70); women: 7 (30) | N/A | Qualitative (motivation to self-monitor and acceptability of the intervention) | 12 months | + |
|  | Donate-Martinez et al [43], 2016, Spain | 74 | Chronic conditions (COPD, type 2 diabetes, and HF) | Manual upload of data on dedicated device or software, 74, mean 67.95 (SD 11.14), men: 49 (66); women: 25 (44) | N/A | Satisfaction and QoL | 12 months | = |
|  | Grady et al [53], 2016, United Kingdom | 40 | Type 1 and 2 diabetes | Manual upload of data on dedicated device or software, 40, median 49.3 (IQR 24-70), men: 18 (45); women: 22 (55) | N/A | HbA1c | 3 months | + |
|  | Amir et al [14], 2017, Israel | 50 | HF | Automated upload of data on dedicated device or software, 50, mean 73.8 (SD 10.3), men: 31 (62); women: 19 (38) | N/A | Number of HF-related hospitalizations | 3 months | + |
|  | Nissen and Lindhardt [82], 2017, Denmark | 14 | COPD | Manual readings via telephone, 14, mean 69.5 (SD not reported), men: 6 (43); women: 8 (57) | N/A | Qualitative (patients’ experience of the intervention) | 6 months | + |
|  | Orozco-Beltran et al [85], 2017, Spain | 521 | Chronic conditions (COPD, type 2 diabetes, and HF) | Manual upload of data on dedicated device or software, 521, mean 70 (SD 10.3), men: 318 (61); women: 203 (39) | N/A | HbA1c, BP, and number of hospital admissions | 12 months | + |
|  | Lee et al [68], 2018, United Kingdom | 10 | Type 2 diabetes | Manual upload of data on dedicated device or software, 10, mean 62.6 (SD not reported), men: 2 (20); women: 8 (80) | N/A | Qualitative (facilitating positive experience and acceptance of telemonitoring) | 1.5 to 3.5 years | + |
|  | Lee et al [67], 2019, Malaysia | 48 | Type 2 diabetes | Manual upload of data on dedicated device or software, 48, mean 51.9 (SD not reported), men: 21 (44); women: 27 (56) | N/A | Qualitative (satisfaction and participants’ perception of telemonitoring) | Not reported | + |
|  | Michaud et al [76], 2018, United States | 955 | Type 2 diabetes | Manual upload of data on dedicated device or software, 955, median 60 (IQR 19-81), men: 432 (45.0); women: 523 (55) | N/A | BP and HbA1c | 3 months | + |
|  | Grant et al [54], 2019, United Kingdom | 40 | Hypertension | Manual upload of data on dedicated device or software, 23, not reported, 45 men; 55 women^b^ | Paper diary, 23, not reported, 45 men; 55 women^b^ | BP | 6 months | + |
|  | Van Berkel et al [102], 2019, United Kingdom | 3562 | Chronic conditions (COPD, type 2 diabetes, and HF) | Manual upload of data on dedicated device or software, 3562, median 66.5 (IQR 66.1-66.9), not reported | N/A | Number of hospital admissions | 12 months | + |
|  | Buis et al [26], 2020, United States | 15 | Hypertension | Real-time home BP tracking app, 15, mean 52.2 (SD 6.0), men: 8 (53); women: 7 (547) | N/A | BP | 12 weeks | + |
|  | Leng Chow et al [69], 2020, Singapore | 205 | HF | Automated upload of data on dedicated device or software, 150, mean 57.9 (SD 12.3), men: 91 (61); women: 59 (39) | Usual care+telephone support, 55, mean 63.9 (SD 14.2), men: 32 (58); women: 23 (42) | ACH and number of HF-related hospitalizations | 12 months | = |
|  | Pekmezaris et al [86], 2020, United States | 12 | Type 2 diabetes | Manual upload of data on dedicated device or software, 12, not reported, not reported | N/A | Qualitative (patients’ acceptability and usability of the device) | 1 month | Several aspects of the intervention to be improved |

^a^BP: blood pressure.

^b^Absolute value not reported in the paper.

^c^Positive impact of telemonitoring over comparator.

^d^HbA1c: glycated hemoglobin.

^e^CHF: congestive heart failure.

^f^ACM: all-cause mortality.

^g^Negative impact of telemonitoring over comparator.

^h^HF: heart failure.

^i^No differences between telemonitoring and usual care.

^j^ACH: all-cause hospitalization.

^k^COPD: chronic obstructive pulmonary disease.

^l^QoL: quality of life.

^m^MLHFQ: Minnesota Living with Heart Failure Questionnaire.

^n^SF-36: Short Form 36 Health Survey Questionnaire.

^o^PHQ: Physical Health Questionnaire.

^p^AF: atrial fibrillation.

^q^GHQ: General Health Questionnaire.

^r^15D: 15-Dimension Instrument of Health-Related Quality of Life.

^s^N/A: not applicable.

^t^H&M: hospitalization and mortality.

^u^SF-12: 12-item Short Form Health Survey.

^v^CRQ: Chronic Respiratory Disease Questionnaire.
